# An open-source framework for physiologically-based pharmacokinetic modeling of kinetics in the female reproductive tract

**DOI:** 10.3389/fphar.2026.1797636

**Published:** 2026-07-02

**Authors:** S. Fischer-Holzhausen, N. Nauwelaerts, L. S. Lautz, S. Mirzaee, V. Baier, S. Schaller, M. Siccardi

**Affiliations:** ESQlabs GmbH, Saterland, Germany

**Keywords:** open systems pharmacology suite, open-source, PBPK, pharmacokinetics, uterine drug delivery, vaginal drug delivery, women’s health

## Abstract

**Introduction:**

The female reproductive tract (FRT) is underrepresented in Physiologically-Based Pharmacokinetic (PBPK) models, creating a critical gap for data-driven, female-specific decision-making. To address this gap, a PBPK model extension for the FRT organs was developed in PK-Sim/MoBi.

**Methods:**

Physiological parameters were derived from a review of data in the public domain. The extension module characterizes four tissue compartments (cervix, vagina, endometrium, and myometrium) and two fluid compartments (uterine and cervicovaginal fluids), and enables the prediction of FRT tissue and plasma concentrations for several routes of administration, including vaginal and intra-uterine.

**Results:**

The model’s predictions for systemic and local drug administration of levonorgestrel and metronidazole were validated against clinical data, demonstrating that the module accurately captures pharmacokinetics across multiple routes of administration.

**Discussion:**

The module can easily be adopted for any PBPK model in the Open Systems Pharmacology Suite. This work makes an important contribution to better representing female physiology in PBPK models and has the potential to improve women’s healthcare.

## 1 Introduction

Medical research and drug development have historically prioritized male physiology, leaving critical gaps in understanding female-specific responses. Adverse events in the 1960s and 1970s related to drugs such as thalidomide and diethylstilbestrol or devices like the Dalkon Shield (Adam et al., 1977; Hoover et al., 2011; Mumford and Kessel, 1992) led to the exclusion of women from clinical trials as an attempt to protect women. Further, concerns about hormonal variability, and the longstanding assumption that data from men can be generalized to women resulted into a bias in medical research and drug development (Merone et al., 2022; Holdcroft, 2007). Since the early 1990s, however, the inclusion of women in clinical trials has been generally required^1^, recognizing that drug pharmacokinetics (PK) and pharmacodynamics can differ between sexes (Pare et al., 2011; Vasisht et al., 2021). Hence, adequate female representation is considered important to detect clinically relevant sex-related differences (Merkatz and Junod, 1994; Gross et al., 2022).

A lack of data stratified by biological sex can compromise drug effectiveness and increase the risk of adverse effects due to physiological variation (Oi Yan Chan et al., 2022). Nevertheless, the hesitations to include women in clinical trials are still present (Rehan et al., 2024). Model-informed drug development can support planning for safer and more targeted trials, making the inclusion of women in early phases both feasible and justified. Additionally, model-informed drug development supports sex-specific dosing strategies and, when combined with *in vitro* methods, helps fill data gaps in situations where direct studies may not be possible.

Physiologically based pharmacokinetic (PBPK) models are a well-established mechanistic modeling framework to predict a compound's absorption, distribution, metabolism, and elimination. They allow the investigation of variability sources originating from physiological and anatomical differences, by population or individual-driven model parameterization^2^ ^,^ ^3^. They have also been applied to study sex-specific risk for chemical exposure (Xiao et al., 2022; Hartmanshenn et al., 2016). Even though PBPK models are well-positioned to perform sex-specific analysis, very few modeling frameworks provide a sex-specific model structure, meaning most models do not represent the tissues of the reproductive organs or the breast.

The female reproductive tract (FRT) consists of external organs (labia majora and minora, clitoris, vaginal opening, hymen and urethra) and internal organs (vagina, cervix, uterus, fallopian tubes, ovaries). It plays key roles in reproduction, hormonal function, and sexual health. The anatomical and physiological features of the female reproductive system give rise to a distinct set of healthcare needs — encompassing vulnerability to infections, conditions such as endometriosis and pelvic inflammatory disease, various cancers, hormone-related disorders, menstrual issues, fertility challenges, and menopausal complications.

**FIGURE 1:**
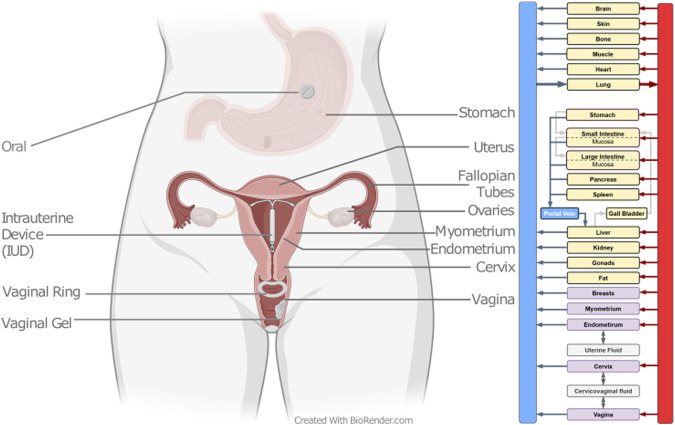
Representation of the Physiologically based Pharmacokinetic model structure. The left figure gives a schematic representation of the organs of the FRT. Furthermore, the different routes of administration that have been included in the exposure simulations are illustrated (Created in BioRender)^8^. The right figure gives a schematic overview of the full-body PBPK model. The presented extension module includes five tissue compartments (endometrium, myometrium, vagina, cervix and breasts), marked in pink, and two fluid compartments (uterine fluid and cervicovaginal fluid), presented in white. The PK-Sim base PBPK model already includes all the remaining organs.

Representing the organs of the female reproductive tract (Figure 1) explicitly in a PBPK model offers several benefits. Firstly, such a model extension enables the prediction of tissue and fluid concentrations within the FRT, after both local and systemic exposure. Secondly, it provides valuable predictions for applications where the FRT is the primary treatment target. Thirdly, establishing tools to support the development of vaginal and uterine drug administration systems—both of which bypass first-pass liver metabolism—can aid drug development and clinical trial design. However, despite the advantages of drug delivery via the FRT (Sanchez Armengol et al., 2025), the development of such local administration systems is hindered by the lack of early drug discovery tools, the high cost of experimental set-ups, ethical concerns, and limited patient consent for clinical studies (Adams et al., 2025).

The literature around FRT PBPK models is limited. (Thakur et al., 2024) and (Kay et al., 2018) published PBPK models that comprehensively described the physiology and anatomy of the female reproductive tract organs. Both studies highlight the utility of these models for conducting cost-effective and accessible *in silico* mechanistic studies of vaginal drug absorption. Despite this potential, the studies also underscore that data gaps remain a major challenge when building such female-tailored PBPK model extensions.

In this work, we developed a PBPK extension module for the female reproductive organs available in the Open Systems Pharmacology (OPS) Suite^4^ and it is available on GitHub^5^. This module can extend any PBPK model available in the OPS Suite by adding the FRT organs. The mechanistic FRT extension module enables the prediction of tissue and plasma concentration following oral and intravenous administration as well as vaginal and intrauterine exposure. It was verified by comparing model predictions to clinical data for levonorgestrel and metronidazole, considering in total four routes of administration (intravenous, oral, vaginal and intrauterine delivery). Predicting drug exposure in female reproductive tract tissues is important for applications such as contraception, reproductive toxicology, and the treatment and prevention of communicable diseases. Hence, a robust extension module as part of a larger modeling ecosystem is expected to enhance the applicability of PBPK models to account for female-specific needs during drug development, clinical trial designs, and risk assessment.

## 2 Methods

### 2.1 Software

PBPK models were developed using PK-Sim® and MoBi® version 12.0^6^. Initial compound models were created in PK-Sim and extended in MoBi to incorporate the developed FRT extension module. Parameter identification was performed in MoBi. Data extraction from the published literature was done using WebPlotDigitizer v4.8. Sensitivity analysis and visualization of simulation results were performed in Claude (Anthropic) was used to assist in the preparation of R code for figure formatting and visualization. Simulations were performed using the CVODE solver with standard solver setting: absolute tolerance of 1E-10 and relative tolerance of 1E-5. The {esqlabsR} package^7^ was used to run simulations, calculate pharmacokinetic parameters and generate graphical outputs.

### 2.2 PBPK model structure

The PBPK model builds up in the standard whole-body PBPK model structure in PK-Sim (Figure 1). The standard human model comprises 18 organs and tissues. Each organ is represented as a separate compartment, which is divided into the following four sub-compartments: plasma, blood cells, interstitial, and intracellular space (Willmann et al., 2003). All absorption, distribution, metabolism and excretion processes are captured by ordinary differential equations (Kuepfer et al., 2016). The PK-Sim parameter database enables model parameterization adjusted to individual characteristics such as age and biological sex. To represent the female anatomy a breast compartment was added during the model extension process.

### 2.3 Female reproductive tract PBPK extension module

The FRT structure is developed as a separate extension module that can be added to any PBPK base model in MoBi version 12.0. The model extension (Figure 1) simplifies the complex anatomy of the FRT, since there was no sufficient data to characterize all tissues of the FRT. The module consists of four FRT tissue compartments (endometrium, myometrium, vagina, and cervix) and two fluid compartments, uterine fluid and cervicovaginal fluid. A breast compartment is also included in the module to improve the overall representation of female physiology, although the focus of this work remains on the organs of the FRT. All tissue compartments have the same sub-compartmentation as the tissues of the base whole-body structure. Each tissue is connected via the systemic circulation, allowing drug exchange between tissues. Direct transfer between the endometrium and myometrium, despite their anatomical adjacency, is neglected due to the lack of data to quantify interlayer permeability.

The whole-body PBPK base model parameters are obtained from the PK-Sim database that provides age and sex-dependent model parameters^9^. A structured literature search was conducted to identify anatomical and physiological data relevant to the female reproductive tract. Studies were included if they reported quantitative measurements in humans, focusing on adult, non-pregnant women. Data from animal studies was not included due to unclear translatability. The derived parameters include compartment volumes and specific blood flow rates, as presented in Table 1. Data for detailed characterization of tissues compositions for the FRT tissues was not available. Therefore, we adopted the parameterization of the muscle compartment for those tissues. The tissue-specific parameterization of breast compartment, referring to the total breast tissue, was adopted from the pregnant population in the OSP Suite database (Dallmann et al., 2017).

**TABLE 1:** Overview of physiological parameters for the female-specific organs of the extension module.

Parameter	Compartment	Mean (Parameter value)	Standard deviation	Unit	References
Tissue volume	Endometrium	0.003	0.0015	L	Karaca-Saygili et al. (2018)
	Myometrium	0.079	0.023	L	Dallmann et al. (2017)
	Cervix	0.025	0.0089	L	Ahmed et al. (2017)
	Vagina	0.047	0.015	L	Calculated using length and tissue thickness
	Breasts	0.71	Not reported	L	Lagend et al. (2018)
Tissue surface area	Endometrium	6	Not reported	cm^2^	Chen et al. (2020), Goldstuck (2018), Feng et al. (2022)
	Cervix	30.2	Not reported	cm^2^	Thakur et al. (2024)
	Vagina	87.5	Not reported	cm^2^	Pendergrass et al. (2003)
Tissue thickness	Endometrium	9	Not reported	mm	Garcia et al. (2022)
	Cervix	16.9	Not reported	mm	Thakur et al. (2024)
	Vagina	2.2	Not reported	mm	Thakur et al. (2024)
Specific blood flow	Endometrium	422.9	21.0	mL/min/100 g organ	Dallmann et al. (2017)
	Myometrium	19.4	0.97	mL/min/100 g organ	Dallmann et al. (2017)
	Cervix	8.7	0.44	mL/min/100 g organ	Assumption
	Vagina	9.8	0.49*	mL/min/100 g organ	Wagner and Ottesen (1980)
	Breasts	2.91	Not reported	mL/min/100 g organ	Dallmann et al. (2017)
Volume	Cervicovaginal fluid	0.0005	Not reported	L	Mitchell et al. (2011)
	Uterine fluid	0.0001	Not reported	L	Lykke et al. (2021)
pH	Cervicovaginal fluid	5	Not reported	​	Lykke et al. (2021)
	Uterine fluid	7.4	Not reported	​	Lykke et al. (2021)

* Assumption based on variability observed for other tissues.

#### 2.3.1 Vaginal and uterine absorption model

Compounds released into the uterine or cervicovaginal fluid enter systemic circulation by crossing the tissues of the FRT. The transport equations for the compound transfer between the uterine fluid and the endometrial tissue, as well as between the cervicovaginal fluid and vaginal and cervical tissue are based on the mathematical description of a diffusion process. The equation describing the amount of compound in the vaginal or uterine fluid $N_{fluid}$ reads:

$$\frac{{dN}_{fluid}}{dt}=D\;\frac{{SA}_{tissue}}{H_{tissue}}\;\left(C_{tissue}\cdot {fu}_{tissue}\cdot scaling-C_{fluid}\cdot f_{union}\right)+\frac{{dN}_{form}}{dt}.$$ (1)

D is the diffusion coefficient in the tissue through which the compound diffuses. Since experimental data on drug transport across tissues of the FRT organs were unavailable, we estimated the diffusion coefficients for the cervix and vagina using a Quantitative Structure-Activity Relationship (QSAR) model developed by (Chen et al., 2015). Although designed initially for skin tissues, we followed the rationale of Thakur et al. assuming its applicability to vaginal tissue due to physiological similarities. The diffusion coefficient for the uptake from the uterine fluid was initially assumed to be equal to the value for cervical and vaginal uptake. However, numerical optimization was needed to ensure that the model captured the clinical data appropriately. The dimensions of the tissues are described by a surface area (SA) and a thickness (H). C denotes the concentration in the tissue and fluid, respectively. The unbound fraction in tissue is denoted by ${fu}_{tissue}$ . As only unionized compounds can diffuse into the tissues, this fraction is captured by $f_{union}$ . The fraction unionized was derived using the Henderson–Hasselbalch equation (Po and Senozan, 2001), which relates the drug’s pKa to the local pH and thus allows calculation of the proportion of molecules in the unionized form.

We also included a scaling factor in the transport formula. It can be interpreted as a correction factor for the unbound fraction in tissue and can be optimized when tissue concentration data is available. By default, this value is set to one but provides one degree of freedom for numerical optimization. In practice, the unbound fraction in tissue is rarely reported. Hence, the parameter is usually derived based on assumptions. The introduced scaling factor can be interpreted as a correction factor for additional and unknown binding in the tissues, which has not been captured when deriving ${fu}_{tissue}$ .

In case of local administration, e.g., an intrauterine device or a vaginal tablet, the source term $\frac{{dN}_{form}}{dt}$ describes the mass transfer from the formulation into the uterine or cervicovaginal fluid. The absorption model presented in Equation 1 assumes that all compartments are well-stirred, and that the fluid-tissue interface is uniform. While $\frac{{dN}_{form}}{dt}$ is zero when working with systemic exposure routes, the compound release $\frac{{dN}_{form}}{dt}$ from an intrauterine or intravaginal formulation to the fluids of the FRT can be formulated with a release rate or a release profile. The most appropriate mathematical expression for the release term depends on the administration route, formulation, and available data. For this work, we evaluated two expressions for $\frac{{dN}_{form}}{dt}$ , namely, a zero-order process with a release rate $k_{release}$ (mg/h):

$$\frac{{dN}_{form}}{dt}=k_{release}$$ (2)

and a Weibull function:

$$\frac{{dN}_{form}}{dt}=1-\exp\left(-{\left(\frac{t}{b}\right)}^{a}\right).$$ (3)

The parameters a and b characterize the shape and the scale of the Weibull release profile. For the levonorgestrel IUD 52 mg longitudinal release data as well as an expected average release rate along with PK data (Cicali et al., 2021) are publicly available. Therefore, we investigated the differences in predicted plasma concentration when using (A) a zero-order release term (Equation 2) and (B) a Weibull release term (Equation 3) for the Levonorgestrel (LNG) IUD 52 mg.

### 2.4 PBPK compound models

The compounds were selected based on the availability of *in vivo* plasma, tissue and fluid concentrations after systemic and local administration to allow the development and evaluation of a base PBPK model. Furthermore, clinical data for the characterization of the different local drug administration formulations needed to be available. The selected compounds were levonorgestrel and metronidazole.

#### 2.4.1 Levonorgestrel

Levonorgestrel is a synthetic progestogen widely used in various hormonal contraceptive formulations. Cicali et al. (2021) developed a PBPK model for orally administered levonorgestrel, available on GitHub. Their model accounts for CYP3A4-mediated metabolism as well as binding to systemic albumin and sex hormone-binding globulin. Their drug-specific PBPK model parameters are summarized in Table 1 in the Supplementary Material.

We used the PBPK model from Cicali et al. as a base model and extended it with our FRT module to predict levonorgestrel concentrations in female reproductive tract tissues following both oral and intrauterine administrations. The simulated dosing strategies were set up to match the study protocols of Cicali et al., Nilsson et al. and Apter et al. Compound-specific PBPK model parameters that were introduced for the FRT extension are provided in Table 2. Thakur et al. (2024) used a QSAR established for skin (Chen et al., 2015) to predict the diffusion coefficient for the vaginal tissue. We extended the application of the QSAR to have an initial parameter estimated for the cervical and uterine tissue, knowing that their rational for its applicability did not hold for uterine tissue. Therefore, to predict the tissue concentration reported by Nilsson et al., the diffusion parameter for the uptake of the uterine tissue was refitted. Furthermore, we calculated the scaling factor of the unbound fraction in endometrial and myometrial tissue. Our interpretation of this scaling factor is that it should account for differences in LNG target availability, as reported for these tissues (Thi et al., 1975). For the intrauterine device containing 63 mg (IUD 1) and 52 mg (IUD 2) LNG, we characterized release functions. A Weibull function could not be characterized for IUD one due to the lack of longitudinal release data. The parameters of the corresponding release terms are also given in Table 2.

**TABLE 2:** Overview of levonorgestrel-specific parameters for the FRT module.

Parameter	Compartment	Parameter value		Unit	References
Diffusion coefficient	Cervix	2.20 E-7		cm^2^/s	Thakur et al. (2024)
	Vagina	2.20 E-7		cm^2^/s	Thakur et al. (2024)
	Uterus	2.46 E-6	2.20 E-7 (initial value)	cm^2^/s	Fitted*
Fraction unionized	Cervix	1		​	Henderson–Hasselbalch equation
	Vagina	1		​	
IUD 1 Zero order release rate	​	0.1		μmol/day	Nil et al. (1982)
IUD 2 Zero order release rate	​	0.06		μmol/day	IUD labeling^10^
IUD 2 Weibull parameter *a*	​	0.98		​	IUD labeling^8^
IUD 2 Weibull parameter *b*	​	7.55		years	IUD labeling^8^
Scaling factor fraction unbound in tissue	Endometrium	0.11	1 (initial value)	​	Fitted*
	Myometrium	0.23	1 (initial value)	​	Fitted*

* Results of a multiple start optimization are presented in Table 6 and Figure 3 in the Supplementary Material.

#### 2.4.2 Metronidazole

Metronidazole is an antibiotic and antiprotozoal medication used to treat a range of infections, including bacterial vaginosis, trichomoniasis and endometritis. A PBPK model for IV administration of metronidazole was taken from the literature (Nil et al., 1982). The calculation methods were Rodgers and Rowlands for partition coefficients and PK-Sim Standard for cellular permeabilities. The model was transferred to PK-Sim/MoBi V12 and extended for oral and vaginal administration. The drug-specific PBPK base model parameters as derived by Dallmann et al. For the oral formulation, a dissolved formulation was applied to the immediate release formulation, while a Weibull was fitted to clinical data for the extended-release formulation. The parameters specifically for oral demonstrations are in Table 3 in the Supplementary Material.

**TABLE 3:** Metronidazole-specific parameters for different administration types. All release profiles are described with Weibull functions.

Administration	Parameter	Compartment	Value	Unit	Data for model parameterization
​	Diffusion coefficient	Cervix	2.30E-6	cm^2^/s	Thakur et al. (2024)
		Vagina	2.30E-6	cm^2^/s	Thakur et al. (2024)
	Fraction unionized	Cervix	1.00	​	Henderson–Hasselbalch equation
		Vagina	1.00	​	
Vaginal gel 37.5 mg	End time	​	114.18	min	Cunningham et al. (1994)
	Fraction dose	​	0.46	​	
Vaginal tablet 500 mg	End time	​	845.28	min	Fredricsson et al. (1987)
	Fraction dose	​	0.18	​	
Vaginal 500 mg	End time	​	88.11	min	Mattila et al. (1983)
	Fraction dose	​	0.20	​	
Vaginal pessary 500 mg	End time	​	208.46	min	Salas-Herrera et al. (1991)
	Fraction dose	​	0.11	​	

### 2.5 Clinical data for model validation

Levonorgestrel and metronidazole were selected as validation compounds because of the availability of plasma and tissue PK data for multiple systemic and local routes of administration. The primary objective of the validation was to evaluate model performance across different administration routes rather than across physicochemical space. It should be noted that generalizability to compounds with markedly different physicochemical properties, such as weak bases with pronounced pH-dependent ionization or highly lipophilic molecules, remains to be established.

#### 2.5.1 Levonorgestrel

To validate the model’s predictions, we collected plasma and tissue levonorgestrel concentrations following both oral and intrauterine administrations. To replicate the results of Cicali et al. (2021), we included their dataset, which reports levonorgestrel plasma concentrations after a 0.09 mg daily oral dose. We also incorporated the study by Nil et al. (1982), which details plasma and tissue (endometrium, myometrium, fat, and breast) concentrations after 7 days of 0.25 mg daily oral administration and 36–49 days of intrauterine administration with a device releasing 30 μg of levonorgestrel per day. Finally, the (Apter et al., 2014) study on levonorgestrel plasma concentrations for various IUD formulations over extended period of time allowed us to explore different mathematical models for drug release from the IUD and their impact on plasma concentration predictions.

#### 2.5.2 Metronidazole

For metronidazole, clinical data were collected from the literature for intravenous, oral and vaginal administration. The PBPK model from Dallmann et al., was first developed and compared to PK data of healthy women after 500 mg IV administration (Houghton et al., 1979), and further evaluated in pregnant women receiving the same dosing regimen (Visser and Hundt, 1984).

### 2.6 PBPK model evaluation

Model performance was assessed by comparing predicted plasma concentration profiles to observed clinical data (Shepard et al., 2015; Kuemmel et al., 2020). Visual comparisons were made by overlaying clinical data with the predictions. The predicted-to-observed ratios were computed, and 2-fold error interval, as commonly applied (van Borselen et al., 2024), was used to assess model acceptability. Furthermore, PK parameters such as C_max_ and AUC_0-t_ were calculated for the model predictions and compared to observed values from the study reports. The (GMFE)

$$GMFE=10^{\frac{\sum \left|\log\left(\frac{P_{pred}}{P_{obs}}\right)\right|}{n}}$$ (4)

was calculated as a quantitative measure to assess model performance based on predicted and observed PK parameters, $P_{pred}$ and $P_{obs}$ respectively. For data set with single point measurements, it is not feasible to calculate C_max_ and AUC_0-t ._ In such cases, the observed and predicted concentration, $C_{obs}$ and $C_{pred}$ respectively, are used to derive the prediction error (PE) in percent

$$PE=\frac{C_{pred}-C_{obs}}{C_{obs}}\;\cdot 100\%.$$ (5)

### 2.7 Sensitivity analysis

A local sensitivity analysis was performed to assess the impact of model input parameters on the model’s predictions and build confidence in our modelling approach. Furthermore, the results can identify where experimental data would most effectively support the modeling process. The analyzed parameters included organ-specific blood flow and organ composition to understand better tissue-specific parameters introduced for the female reproductive tract organs. Parameters essential for vaginal and uterine absorption models were also evaluated. In total, 40 parameters have been analyzed, and a complete parameter list is provided in Table 5 in the Supplementary Material. The effects on the predicted time curves as well as PK parameters (AUC, C_max_, t_max_) for plasma and tissue (breast, vaginal, cervical, uterine, myometrium and endometrium) concentration were considered in the sensitivity analysis.

The analysis was carried out using the default implementation of a local Sensitivity analysis in {esqlabsR} package.

### 2.8 Population simulations

Exploratory population simulations were performed to assess the impact of variability in the female population. Female European populations (n = 100) were created to match study demographics for age, weight and height. If no data was available, a population was created with age ranging between 29 and 31 years, incorporating the default population variability in OSP. Furthermore, additional variability was implemented for the FRT organs’ volume and specific blood flow. Figure 1 shows the mean and standard deviation for organ volume and specific blood flow collected from literature. A lognormal distribution was assumed for all FRT parameters in the population.

## 3 Results

The established PBPK models were applied to predict levonorgestrel and metronidazole concentrations in plasma and tissues following both systemic and local administrations. Additionally, we evaluated how the selection of the drug release function influences exposure predictions. Lastly, a local sensitivity analysis was conducted to explore the impact of tissue-specific parameters and parameters of the vaginal/uterine absorption model on model outcomes.

### 3.1 Levonorgestrel

The parameters of the base PBPK model were not refined during model extension. We, however, performed model fitting for the parameters of the uterine absorption process. Table 2 provides an overview of the absorption process parameters presenting their initial and optimized values. The uterine diffusion coefficient was refitted using concentration data from endometrium and myometrium. A bracketing uncertainty analysis for the uterine and oral LNG administration (±1 log_10_ around the QSAR-derived estimate) was performed to assess the impact of QSAR transferability uncertainty on model predictions (Figures 4, 5 in the Supplementary Material). For oral administration, the diffusion coefficients have no significant impact on exposure predictions, as systemic exposure is driven by blood flow. For intrauterine administration, the bracketing analysis revealed asymmetric uncertainty for the uterine diffusion coefficient. The upper bound had a modest impact on predicted tissue concentrations, while the lower bound resulted in substantially reduced predictions.

**FIGURE 2:**
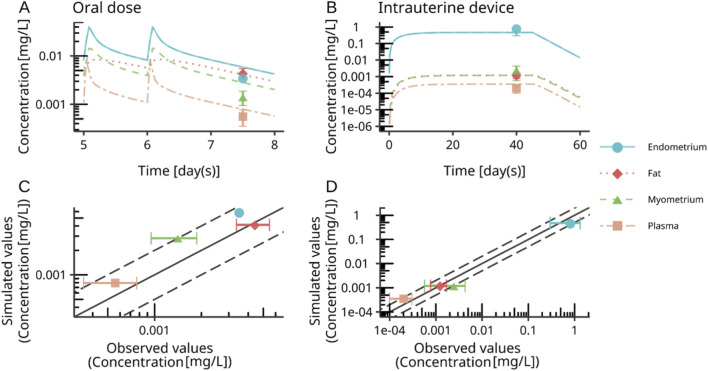
Simulated concentration–time profiles following **(A)** daily oral administration of levonorgestrel (250 μg) and **(B)** intrauterine device administration with a daily release rate of 30 μg. Observed plasma and tissue concentrations reported by Nilson et al. are shown as symbols. Solid and dashed lines represent model-predicted concentrations, with colors corresponding to the respective observed data. Observed versus predicted concentrations for the oral formulation are shown in **(C)**, and corresponding results for the IUD are presented in **(D)**.

The physiological parameters of the base PBPK model were those of a 30-year-old healthy female individual as characterized in the PK-Sim database. The mean parameters for the organs of the female reproductive tract are given in Table 1. The predicted plasma and FRT tissue concentrations following oral (Figure 2A) and IUD (Figure 2B) administration are presented in Figure 2. The predicted vs. observed plots (Figures 2C,D) visually indicate that the model fits the data well. To quantify the model prediction capabilities, the prediction error (PE) (Equation 5) was calculated for the eight observation-prediction pairs (Table 4). Six out of eight PE were under 50%, indicating a very good prediction and two had a PE under 100% marking a good prediction. The direction of the PE indicated that the model tends to slightly underpredict. Due to the sparsity of the data, it was not feasible to calculate the AUC or C_max._ Hence, the GMFE cannot be provided.

**TABLE 4:** Summary of observed and corresponding predicted concentrations for plasma, fat tissue, endometrium and myometrium after oral and IUD administration of LNG, along with their prediction error.

Formulation	Compartment	Measurement time point	Observed concentration^11^ [mg/L]	Predicted concentration [mg/L]	Prediction error [%]
Oral	Plasma	Day 7	7.8E-4	5.6E-04	−28
	Fat		4.6E-3	3.1E-03	−23
	Myometrium		2.8E-3	1.4E-03	−50
	Endometrium		5.6E-3	3.1E-03	−44
IUD	Plasma	Day 36–49 (assumed day 40 for simulation)	1.9E-4	3.4E-04	83
	Fat		1.3E-3	1.2E-03	−3
	Myometrium		2.4E-3	1.2E-03	−51
	Endometrium		7.8E-1	4.7E-01	−40

To capture the tissue concentration in both the endometrium and myometrium, the scaling factor introduced in Equation 1 was optimized. The fitted values were 0.11 for the endometrium and 0.23 for the myometrium, resulting in reduced availability of freely moving levonorgestrel in both tissues. The most significant reduction can be observed in the endometrium. This is qualitatively in line with experimental observations, which suggest that progesterone receptor abundance is higher in the endometrium compared to the myometrium (Thi et al., 1975). Furthermore, these findings align with our hypothesis that the scaling factor accounts for differences in target availability between tissues.

#### 3.1.1 IUD release description

The release from a drug administration device, such as an intrauterine device, can be described mathematically in different ways. The choice of release model depends primarily on the available device-specific release data and clinical data for model calibration. For the IUD 52 mg LNG, we compared two release models: (A) a zero-order release and (B) a Weibull function. The Weibull parameters (Table 2) were obtained by fitting the function to reported release data (see Supplementary Figure 2). In both cases, we used the release parameters derived from the reported release experiments without further refinement. The objective was not to identify an optimal release model, but to evaluate how different mathematical descriptions of device release influence simulated plasma concentration, and to identify where current approaches perform adequately and where refinement would be needed in future work.

**FIGURE 3:**
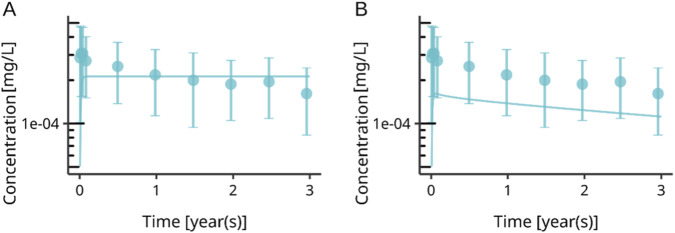
Simulated plasma concentration–time profiles of levonorgestrel (LNG) following intrauterine device (IUD) administration, comparing two different release models: **(A)** zero-order release, assuming a constant daily release rate, and **(B)** Weibull release function, representing a time-dependent, decreasing release rate.

Figure 3 shows the predicted plasma concentration–time profiles for the two release models. Visual inspection suggests that the Weibull function captures the overall shape of the release profile better than the zero-order model, particularly the initial rise and the gradual decline in concentrations over time (Figure 3B). However, pharmacokinetic parameters (Table 5) indicate that the zero-order model better predicts overall drug exposure (AUC). The Weibull model, parameterized from fitting to estimated *in vivo* release rates (Mirena Labeling), systematically underpredicts plasma concentrations resulting in an underestimated AUC. Refining the Weibull parameters could improve this discrepancy. Neither release model predicted *t*_max_ accurately; the zero-order model approximated *C*_max_ but at an incorrect time point.

**TABLE 5:** Summary of observed and predicted LNG plasma concentration after IUD administration.

PK parameter	Observed value (Apter et al., 2014)	Predicted value		GMF (Equation 4)	
​	​	Zero order release	Weibull release	Zero order release	Weibull release
AUC(0–3years) [mg∙h/L]	9.9E-3	9.6E-3	6.0E-3	1.03	1.65
C_max_ [mg/L]	3.4E-4	2.1E-3	1.6E-4	1.62	2.13
t_max_ [days]	14	70.4	22.25	10.06	3.18

The release from the device was described by (A) a zero-order release term and (B) a Weibull function. The impact of the release term on the prediction accuracy is captured by corresponding GMFE.

In conclusion, the Weibull function leads to a better description of the qualitative feature of the release profile. However, in scenario where one is interested in exposure over longer periods, the zero order release term may be sufficient.

### 3.2 Metronidazole

**FIGURE 4:**
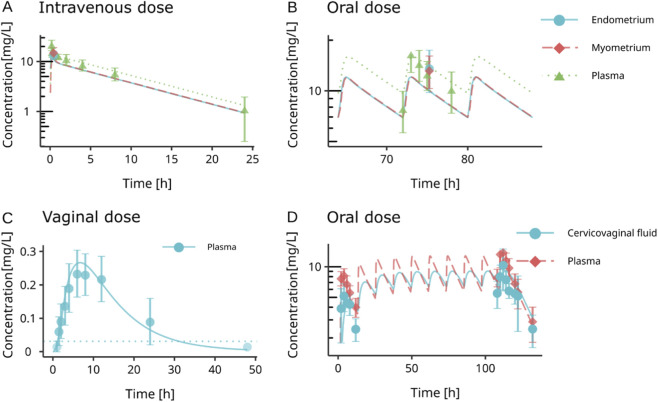
Simulated concentration–time profiles for metronidazole following **(A)** a single intravenous administration of 500 mg **(B)** 400 mg thrice daily oral administration **(C)** single vaginal gel administration of 37.5 mg and **(D)** 400 mg twice daily oral administration. Mean observed plasma and tissue concentrations are shown as symbols, with error bars representing the standard deviation. Solid and dashed lines represent simulated concentrations, with colors corresponding to the respective plasma or tissue concentrations.

Figure 4 shows the concentration-time profiles for metronidazole after intravenous, oral and vaginal administration. The predicted versus observed plots are included in the Supplementary Material. The GMFE on the Cmax in plasma was 1.39, 1.11 and 1.11 for intravenous (n = 3), oral (n = 6) and vaginal (n = 5) administration respectively. The GMFE on the AUC in plasma was 1.18, 1.10 and 1.14 for intravenous, oral and vaginal administration respectively. The prediction error for metronidazole concentration in myometrium and endometrium were −6% and −12% for intravenous and −24% and −27% for oral administration, respectively. PK parameters (AUC and Cmax) are listed in Table 6, including predicted/observed ratio.

**TABLE 6:** Summary of calculated PK parameters for metronidazole.

Scenario	Dose	Route	Compartment	Time range (h)	Cmax (mg/L) (predicted)	AUC (mg*h/L) (predicted)	Cmax (mg/L) (observed)	AUC (mg*h/L) (observed)	Cmax ratio	AUC ratio
Männistö 1984 (+ single points endometrium and myometrium)	500 mg	IV	Plasma	0–24	19.00	128.37	21.58	126.24	0.88	1.02
	400 mg TID	PO	Plasma	72–78	15.95	81.39	16.45	74.92	0.97	1.09
Salas-Herera 1991a	400 mg BID	PO	Plasma	0–12	7.45	57.23	8.27	70.62	0.90	0.81
				108–132	12.80	159.43	13.90	184.40	0.92	0.86
			CVF	0–12	4.45	37.71	5.21	45.16	0.85	0.84
				108–132	9.06	155.11	10.44	135.29	0.87	1.15
Salas-Herrera 1991b	500 mg BID	Vaginal	Plasma	0–12	0.86	7.26	0.80	6.58	1.07	1.10
				96–120	1.43	22.45	1.39	23.56	1.03	0.95
Cunningham 1994	500 mg	PO	Plasma	0–48	9.65	130.39	11.51	138.62	0.84	0.94
	37.5 mg	Vaginal	Plasma	0–48	0.27	4.43	0.23	5.23	1.14	0.85
Matilla 1983	500 mg	IV	Plasma	0–24	16.26	117.81	9.00	90.68	1.81	1.30
	500 mg	PO	Plasma	0–48	7.46	122.50	8.36	119.46	0.89	1.03
	500 mg	Vaginal	Plasma	0–48	1.65	27.72	1.68	33.66	0.98	0.82
Fredericsson 1987	500 mg	IV	Plasma	0–24	23.22	134.37	30.21	106.99	0.77	1.26
	400 mg	PO	Plasma	0–24	7.95	93.12	8.80	90.40	0.90	1.03
	500 mg	Vaginal	Plasma	0–48	1.21	24.59	0.91	21.52	1.33	1.44

### 3.3 Sensitivity analysis

**FIGURE 5:**
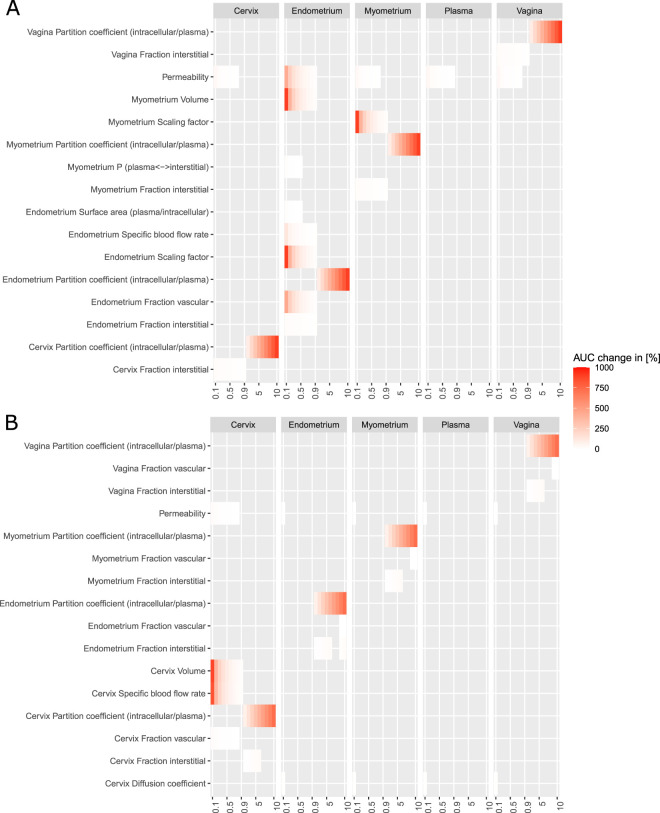
Results of the local sensitivity analysis for AUC. **(A)** Shows the results for the levonorgestrel model following uterine administration and **(B)** For metronidazole following vaginal administration. Only parameters leading to at least 1% change in AUC are presented. The investigated parameters were scaled with a factor in the range [0.1–10.0]. Color intensity indicates the magnitude of AUC change in percent.

Using local sensitivity analysis, we investigated the model parameters’ impact on AUC, C_max_ and t_max_ after uterine LNG and vaginal MET administration. Figure 5 shows an overview of the most sensitive parameters for AUC following local drug administration levonorgestrel (A) and metronidazole (B).

For vaginal administration, the most sensitive parameters relate to the vaginal and cervix tissues, while for uterine administration endometrial and myometrial parameters have a noticeable impact on model predictions.

The analysis of the other PK parameters leads to the same conclusion.

### 3.4 Population simulation

**FIGURE 6:**
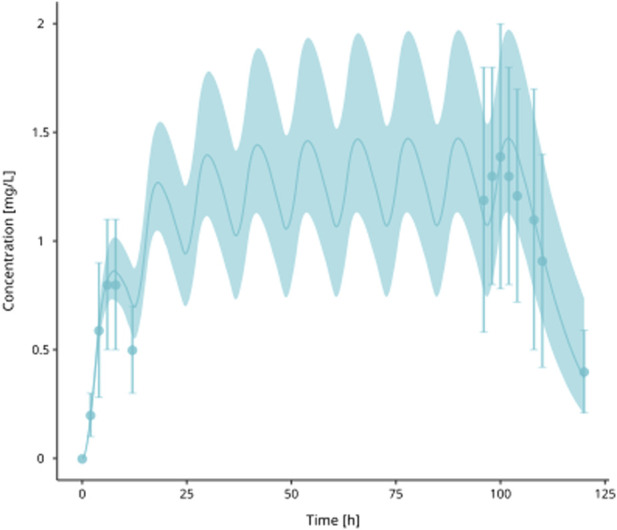
Population simulation. A population simulation (n = 100) was performed for vaginal application of metronidazole 500 mg twice daily vaginal. The line represents the median plasma concentration and shaded area represents 5th to 95th percentile. Points show the data from Salas-Herrera 1991b.

A representative population simulation for a female European population (n = 100) is shown in Figure 6. The simulation was performed for a population with age ranging between 22 and 31 years, and weight varying between 51 and 76 kg according to the study demographics.

## 4 Shiny app for interactive exploration

A Shiny application (available at https://femsim.esqlabs.com/) was developed as a user-friendly and interactive tool for exploring the Female Reproductive Tract PBPK model, eliminating the need for prior experience with PBPK software. The app allows users to simulate local FRT concentrations for levonorgestrel and metronidazole, adjust dosing scenarios, and visualize predicted exposure in key reproductive tissues and compartments. The user can see the differences of simulated concentrations on the selected tissues over time for different administrations within all possible combinations of tissues and selected administrations for each drug.

The FemSim Shiny App, is dependent on the {esqlabsR} package, for simulating the different scenarios selected by the user. It records no data from the user, and all the calculations are done on the fly (within the RAM). Once the calculation is complete, the {dygraphs} package^12^ is used to plot the concentrations on different tissues over time within an interactive plot, where users can select different tissues after different drug administrations, change the Y-axis units and zoom in to any time of interest.

## 5 Discussion

Predicting drug exposure in female reproductive tract tissues is important in drug development, with applications in contraception, reproductive toxicology, and the treatment and prevention of communicable diseases. The physiology of the FRT organs, such as tissue volume, blood flow, and local pH, can influence how a compound is absorbed and distributed, making a mechanistic modeling framework like PBPK well suited to describe tissue pharmacokinetics based on organ-specific characteristics. Here, we present a publicly available PBPK model extension applicable to all PBPK models in PK-Sim/MoBi version 12.0, evaluated with levonorgestrel and metronidazole across at least two routes of administration each. The model was able to capture the pharmacokinetics of both compounds across different routes of administration well. The following discussion addresses the key assumptions, limitations, and areas for future refinement.

We parameterized the extension module using publicly available data, focusing on quantitative data originating from human studies, and published PBPK models including the FRT organs from other PBPK software (Thakur et al., 2024; Kay et al., 2018). As Table 1 indicates by the number of references supporting model parameters, there are generally few sources available to parametrize the model compartments. Furthermore, several sources did not provide sufficient information to characterize parameter variability, which limits the quantification of parameter uncertainty and represents a drawback for population simulation. Lastly, adequate tissue composition data were not available to characterize the endometrium, vagina, and cervix, leading to the use of muscle as a template organ. This assumption directly impacts the calculated partition coefficients for these tissues. To assess the influence of this uncertainty on model output, partition coefficients for all three organs were included in the local sensitivity analysis, which confirmed that they can impact model predictions. Based on these results, expected variation in tissue composition will affect predicted exposure in the FRT compartments. This therefore represents a source of model uncertainty that could be reduced by closing this data gap.

The model does not include the fallopian tubes or ovaries as explicit compartments, due to insufficient human data in the public domain to support parameterization. Given the lack of human data, one could explore the data availability from non-human primates and evaluate their applicability to parameterize a human model.

The sensitivity analysis identified vaginal and cervical tissue parameters as most influential for vaginal administration, and endometrial and myometrial parameters for uterine administration. This is relevant in the context of prediction uncertainty: parameters identified as highly sensitive that are insufficiently supported by data (see Table 1) represent the primary source of model uncertainty, and targeted experimental data collection for these parameters would be the most effective way to reduce it. Specifically, the model would benefit from data to characterize the cervical blood flow as well as data that allows a better characterisation of the tissue properties of the different tissues of the FRT.

The current model parameterization captures the characteristics of a healthy female of child-bearing age best, but it does not incorporate menstrual cycle–driven changes in tissue volume or blood flow. Given the results of our sensitivity analysis, including menstrual cycle-driven changes would add value for certain applications. For example, changes in the endometrium volume and the uterine blood flow could improve certainty for uterine drug delivery systems.

Overall, the data used to derive the physiological parameters presented in Table 1 was quite variable and the model parameterization could be further refined with additional data. The fluid volumes and their pH are assumed to be constant. Hence, the pH gradient observed for the cervicovaginal fluid is neglected. For levonorgestrel and metronidazole, this simplification did not have a significant effect on the fraction of unionized drug available for uptake from the vaginal fluid. For compounds where the unbound fraction is more sensitive to the pH range of the vaginal tract, sub-compartmentalization should be considered. Assuming a constant fluid volume rather than a dynamic turnover description is a further simplification, as secretion and drainage processes influence PK. This limitation is most consequential for drugs with short therapeutic windows or dosing regimens sensitive to the timing of luminal clearance. Calibrating a turnover rate would require additional data such as secretion rate measurements and longitudinal cervical-vaginal fluid concentration profiles with sufficient resolution to separate physical washout from mucosal absorption. Nevertheless, given the modular model development concept, these parameters and mechanisms can be updated as data become available.

A major challenge in model development was the parameterization of drug transfer from vaginal and uterine fluids to target tissues. Ideally, *in vivo* release data would be used to parameterize this process, but in the absence of such data, *in vitro* release data generated under biorelevant conditions could serve as an alternative. However, an established framework for in vitro–in vivo extrapolation (IVIVE) for vaginal and uterine formulations is currently lacking. A QSAR derived for skin tissues was used for the parameterization of diffusion coefficients for the FRT compartments. While this represents the best available approximation in the absence of a tissue-specific QSAR framework, the application is not ideal given the differences between the tissues.

The uterine diffusion coefficient was refitted to available concentration data, which partially mitigates the QSAR limitation for that compartment. We adopted a mixed approach, combining data-driven model fitting with *a priori* knowledge. A key challenge was the incompleteness of publicly available datasets. Notably, the limitation in dataset reporting plasma, tissue, and fluid concentrations simultaneously hindered fully data-driven optimization and has direct implications for parameter identifiability. In the absorption equation, the diffusion coefficient and the scaling factor serve structurally distinct roles, D governs the overall magnitude of flux symmetrically, whereas the scaling factor applies exclusively to the tissue-side driving force, and surface area and membrane thickness were fixed based on literature morphometrics. Nevertheless, fitting both D and the scaling factor to limited concentration data introduces a source of non-identifiability, and alternative parameter sets may describe the data equally well. The fitted parameters should therefore be interpreted as consistent with the available data rather than as uniquely determined mechanistic values.

Alongside quantitative data, we leveraged qualitative insights to inform and constrain the model. For example, the scaling factor introduced to account for LNG availability in the endometrium and myometrium was supported by animal studies showing higher progesterone receptor prevalence in the endometrium. Our fitted value aligned with this, indicating a smaller scaling factor for the endometrium, consistent with a larger unbound fraction of LNG and potentially greater receptor binding in that tissue. In conclusion, despite data limitations and the inherent constraints on parameter identifiability, the simulation results indicate that this mixed approach was an appropriate and pragmatic strategy for parameterizing the model extension. Overall, closing identified data gaps for tissue characteristics, populations information and local transport information would substantially reduce this uncertainty in future work.

Similarly, data to parameterize the vaginal formulation of metronidazole were not available from the literature. Therefore, various vaginal applications of metronidazole were modelled assuming zero-order release kinetics, and automated parameter identification was applied. Furthermore, the administered dose was scaled to align the PBPK model predictions with observed clinical bioavailability. The reduced apparent exposure can be partially explained by the self-cleansing action of the vaginal environment, which promotes drug removal from the administration site. Nonetheless, other less-understood physiological (e.g., local metabolism in the female reproductive tract) or formulation-related effects likely play a role, indicating the need for a better understanding of the female reproductive tract. For the intravenous scenarios, some larger deviations in Cmax were observed, which may reflect uncertainties related to sampling design, formulation assumptions, and tissue partitioning rather than fundamental model misspecification.

The Shiny app offers a practical way for researchers, students, and developers to engage with the model immediately and test different assumptions or use cases. By lowering technical barriers and enabling rapid experimentation, it complements the webinar and online training modules and helps broaden the community of users able to work with the reproductive tract model. It also supports early hypothesis generation, teaching, and potential research extensions, reinforcing the long-term value of an openly accessible modelling framework.

To conclude, a mechanistically informed extension module for the female reproductive tract was developed and implemented within the Open Systems Pharmacology PBPK framework. Integration of this module into existing PBPK models for levonorgestrel and metronidazole enables descriptions of pharmacokinetics following both local and systemic administration. The module is openly available to the scientific community via GitHub, and a user-friendly Shiny app allows exploration of plasma and tissue concentrations for these drugs with minimal technical requirements. Overall, the presented work provides a valuable resource to address female-specific considerations in drug development, clinical trial design, and risk assessment. Valuable model extensions for future work include broadening the chemical space the model is applied to, incorporating mechanistic detail such as dynamic fluid turnover, drug-protein binding and pH stratification in the vaginal tract, and accounting for cycle-driven variability and population-specific parameterizations, for example, in pregnant and menopausal women.

## Data Availability

The original contributions presented in the study are included in the article/Supplementary Material, further inquiries can be directed to the corresponding author.

## Footnotes

1 ICH. Gender considerations in the conduct of clinical trials. 2005;

2 OECD. 2021. *Guidance Document on the Characterisation, Validation and Reporting of Physiologically Based Kinetic (PBK) Models for Regulatory Purposes*. OECD Series on Testing and Assessment. OECD. https://doi.org/10.1787/d0de241f-en.

3 WHO, International Programme on Chemical Safety, and Inter-Organization Programme for the Sound Management of Chemicals, eds. 2010. *Characterization and Application of Physiologically Based Pharmacokinetic Models in Risk Assessment*. Harmonization Project Document, No. 9. World Health Organization.

4
http://www.open-systems-pharmacology.org/.

5
https://github.com/Open-Systems-Pharmacology/Female-Reproductive-Tract-PBPK-Module.

6
https://github.com/Open-Systems-Pharmacology/Suite/releases.

7 ESQlabs GmbH. 2025. *ESQlabsR*. R. V. 5.5.0. Released August. https://esqlabs.github.io/esqlabsR/.

8 Created in BioRender. Albrecht, M. (2026) https://BioRender.com/6xsupvq.

9
https://docs.open-systems-pharmacology.org/working-with-pk-sim/pk-sim-documentation/pk-sim-creating-individuals.

10
https://labeling.bayerhealthcare.com/html/products/pi/Mirena_PI.pdf

11 converted from reported data using the assumption for tissue density of 1 g/L

12 Vanderkam, Dan, J. J. Allaire, Jonathan Owen, Daniel Gromer, and Benoit Thieurmel. 2023. *Dygraphs: Interface to “Dygraphs” Interactive Time Series Charting Library*. https://github.com/rstudio/dygraphs.
