# Supplementary material for: An open-source framework for physiologically-based pharmacokinetic modeling of kinetics in the female reproductive tract
Source: Front Pharmacol. 2026 Jul 2;17:1797636. doi: 10.3389/fphar.2026.1797636 (PMC13373587; doi:10.3389/fphar.2026.1797636)
Supplement: Supplementary file 1 [file Supplementaryfile1.pdf]

Supplementary Material

Table 1: Levonorgestrel PBPK model parameters.

Parameter	Value	Unit	Reference
Molecular weight	312.4	g/mol	(Cicali et al. 2021)
Lipophilicity	4.00		(Cicali et al. 2021)
Albumin binding parameter K_{off}	6.5	1/min	(Cicali et al. 2021)
Albumin binding parameter K_d	6	µmol/L	(Cicali et al. 2021)
SHBG binding parameter K_{off}	17.4	1/min	(Cicali et al. 2021)
SHBG binding parameter K_d	0.0005	µmol/L	(Cicali et al. 2021)
Aqueous solubility	2.05	mg/L	(Cicali et al. 2021)
FaSSIF solubility	14.0	mg/L at pH 6.5	(Cicali et al. 2021)
$Cl_{specific}$/CYP3A4	1.45	µL/min/pmol CYP3A4	(Cicali et al. 2021)
Other hepatic clearance	0.1	L/h/kg	(Cicali et al. 2021)
Specific intestinal permeability	0.0002	cm/min	(Cicali et al. 2021)
Dissolution time	30	min	(Cicali et al. 2021)

Table 2: Metronidazole PBPK model parameters.

Parameter	Value	Unit	Reference
Molecular weight	171.00	g/mol	(Dallmann et al. 2018)
pKa (compound type)	2.49 (base)		(Dallmann et al. 2018)
Fraction unbound	0.89		(Dallmann et al. 2018)
Plasma protein binding partner	Albumin		(Dallmann et al. 2018)
Lipophilicity	-0.02		(Dallmann et al. 2018)
Solubility (reference pH)	9500.00 (7.00)	mg/L	Assumption<br>(Dallmann et al. 2018)
CYP2A6 in vitro metabolic rate in the presence of liver microsomes – Michaelis-Menten<br>- Km<br>- kcat	 0.38<br>1.45	 mmol/L<br>1/min	(Pearce et al. 2013)<br>(Dallmann et al. 2018)
CYP3A4 in vitro metabolic rat in the presence of liver microsomes – Michaelis-Menten<br>- Km<br>- kcat	 33.10<br>72.46	 mmol/L<br>1/min	(Pearce et al. 2013)<br>(Dallmann et al. 2018)
Total hepatic clearance – Liver Plasma Clearance – specific clearance	0.013	1/min	(Dallmann et al. 2018)
Renal clearances – Kidney Plasma	0.022	1/min	(Dallmann et al. 2018)

Clearance – Specific clearance			

Table 3: Oral formulation parameters for metronidazole.

Parameter	Value	Unit	Reference
Specific intestinal permeability	4.32E-6<br><br>(initial value 1.36E-6)	cm/min	Parameter identification based on (Männistö et al. 1984) (PO 400 mg TID) and (Salas-Herrera et al. 1991) (PO 400 mg BID)
Dissolution time (50% dissolved)	87.08	min	Parameter identification based on (Männistö et al. 1984) (PO 400 mg TID) and (Salas-Herrera et al. 1991) (PO 400 mg BID)
Dissolution shape	51.56		
Lag time	0	min	Assumption

Table 4: Overview of clinical studies for metronidazole.

Reference	Administration route	Dose	Clinical data	Population *	Weight (kg)	Height (cm)	Age (years)	Number of participants
(Männistö et al. 1984)	IV (20 min)	500 mg	Plasma Endometrium Myometrium Ovarium Tuba	European ICRP (female)	64.6	160	48.9	20
(Salas-Herrera et al. 1991)	PO	400 mg TID		European ICRP (female)	64.8	163.1	47.5	10
	PO	400 mg BID	Plasma Cervicovaginal fluid	European ICRP (female)	62.8	NA	25.3	6
(Cunningham et al. 1994)	PO	500 mg	Plasma	European ICRP (female)	59.1	NA	27.4	12
	Vaginal gel	37.5 mg						
(Fredricsson et al. 1987)	IV (15 min)	500 mg	Plasma	European ICRP (female)	22.5	NA	NA	5
	PO	500 mg						
	Vaginal tablet	500 mg						
(Salas-Herrera et al. 1991)	Vaginal pessary	500 mg BID	Plasma	European ICRP (female)	60.2	NA	25.3	12

(Mattila et al. 1983)	IV (20 min)	500 mg	Plasma	European ICRP (male)	77	180	22	6
	PO	500 mg		European ICRP (1 male/ 8 females)	77	178	22	9
	Vaginal	500 mg		European ICRP (female)	58	165	23	6

Abbreviations: NA: not available; PO: per os ; IV: intravenous; TID: thrice daily; BID: bidaily
* European ICRP population was assumed when no information about the population was reported

Table 5: Overview of parameters selected for the sensitivity analysis.

Parameter	Compartment
Partition coefficient (intracellular/plasma)	Vagina
	Endometrium
	Myometrium
	Cervix
Surface area (interstitial/intracellular)	Vagina
	Endometrium
	Myometrium
	Cervix
Surface area (plasma/interstitial)	Vagina
	Endometrium
	Myometrium
	Cervix
Fraction vascular	Vagina
	Endometrium
	Myometrium

	Cervix
Fraction interstitial	Vagina
	Endometrium
	Myometrium
	Cervix
Specific blood flow rate	Vagina
	Endometrium
	Myometrium
	Cervix
Volume	Vagina
	Endometrium
	Myometrium
	Cervix
Partition coefficient (plasma<->interstitial)	Vagina
	Endometrium
	Myometrium
	Cervix
Molecule permeability	

Diffusion coefficient	Endometrium
	Cervix
	Vagina

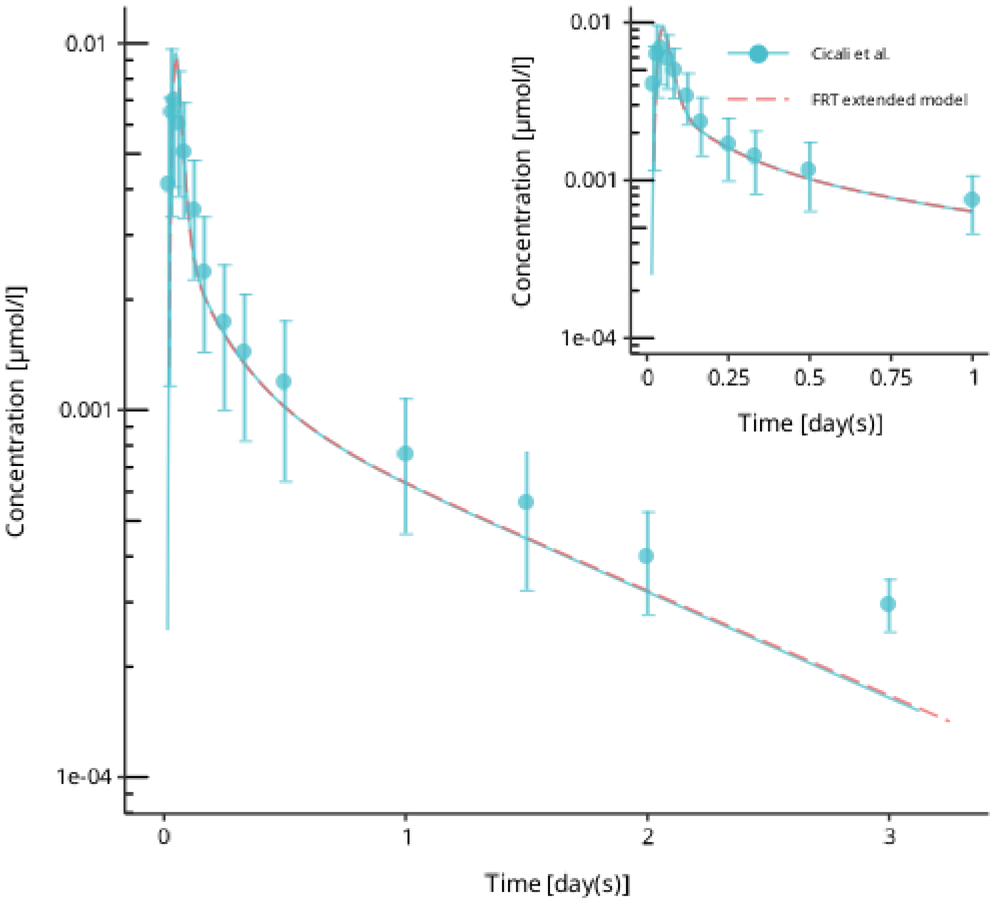

Figure 1: Comparison between Cicali et al. 2021 model predictions and predictions of the PBPK model including organs of the FRT.

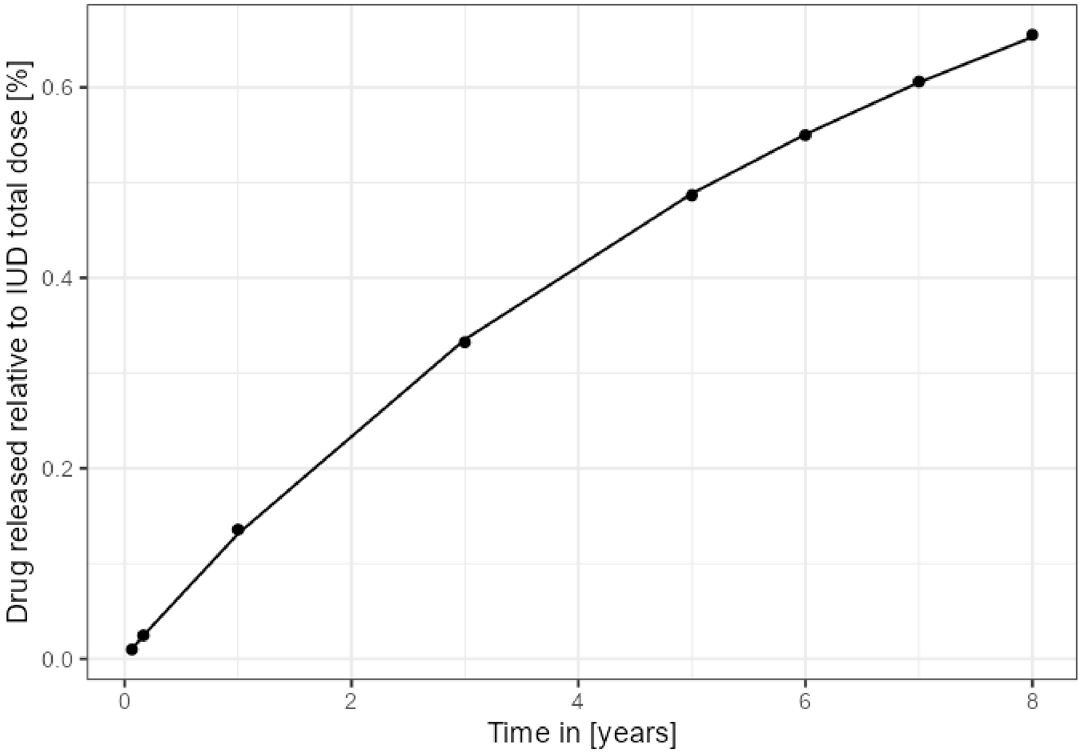

Figure 2: Estimated in-vivo release from the IUD as fraction of total dose over time presented as points. The line presented the fitted Weibull function.

The parameters of the Weibull function, describing drug release from IUD 52mg LNG, were established using estimated in vivo release rates for device.
The derived Weibull function reads:

$$W(t) = \mathbf{1} - \boldsymbol{exp}\left(-\left(\frac{t}{\mathbf{7.55}}\right)^{\mathbf{0.97}}\right).$$

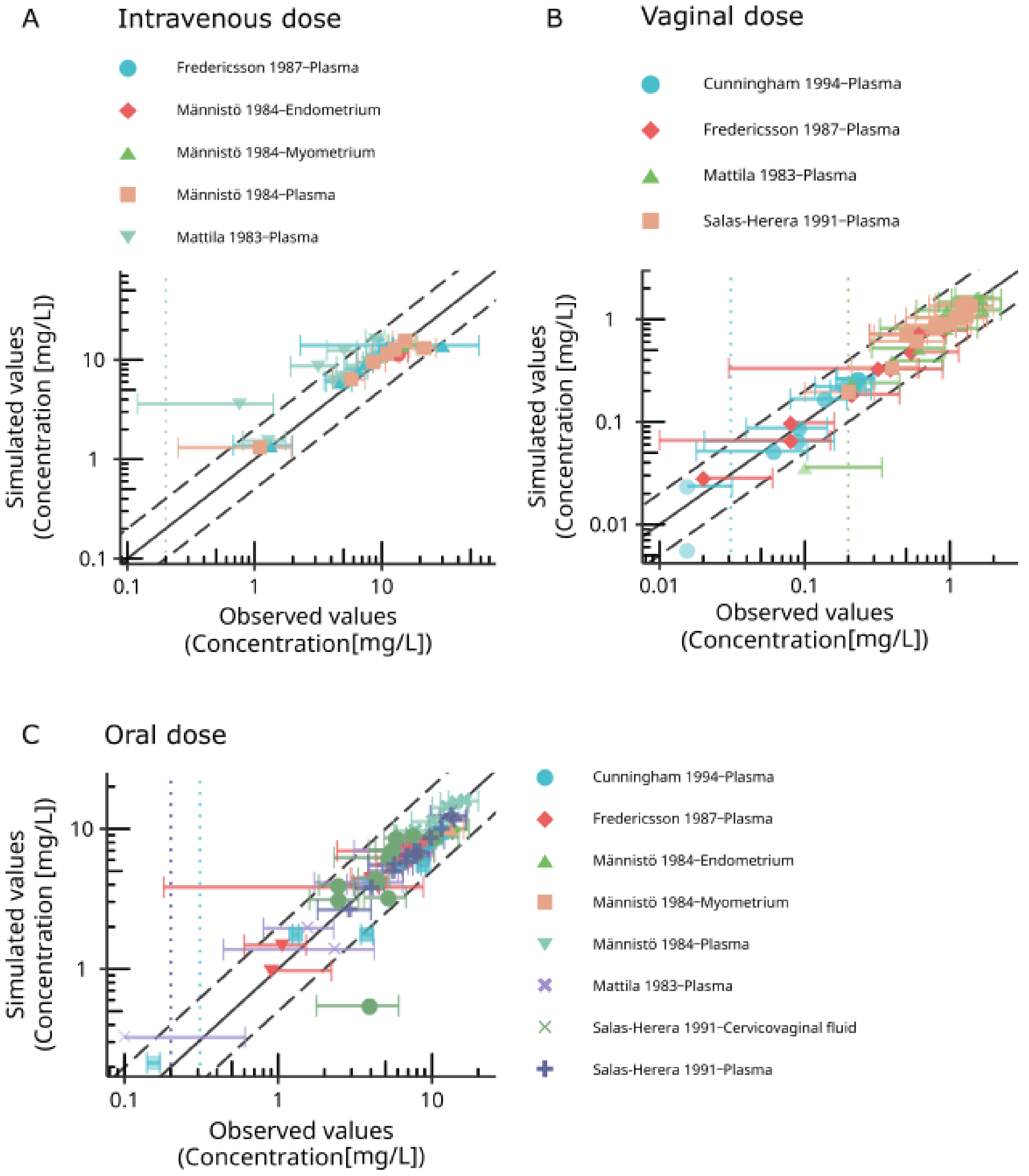

Figure 3: Predicted versus observed plot for metronidazole
Predicted versus observed concentrations for different studies and organs are shown for intravenous (a), vaginal (b) and oral (c) administration. Points represent mean concentrations with standard deviation shown as error bars. Solid line represents unity; dashed lines represent 2-fold deviation

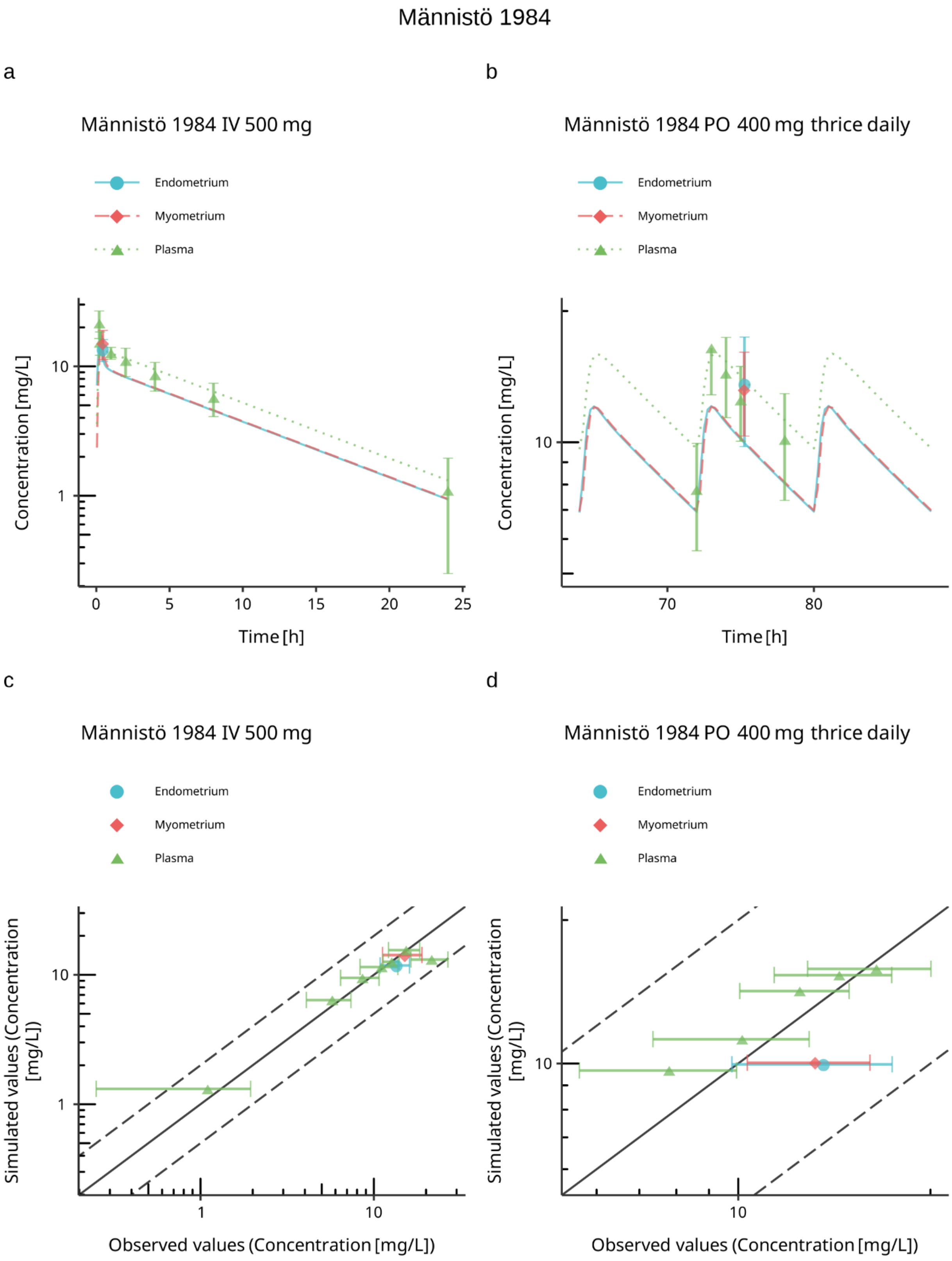

Figure 4: Model prediction for Männistö et al. 1984 study.

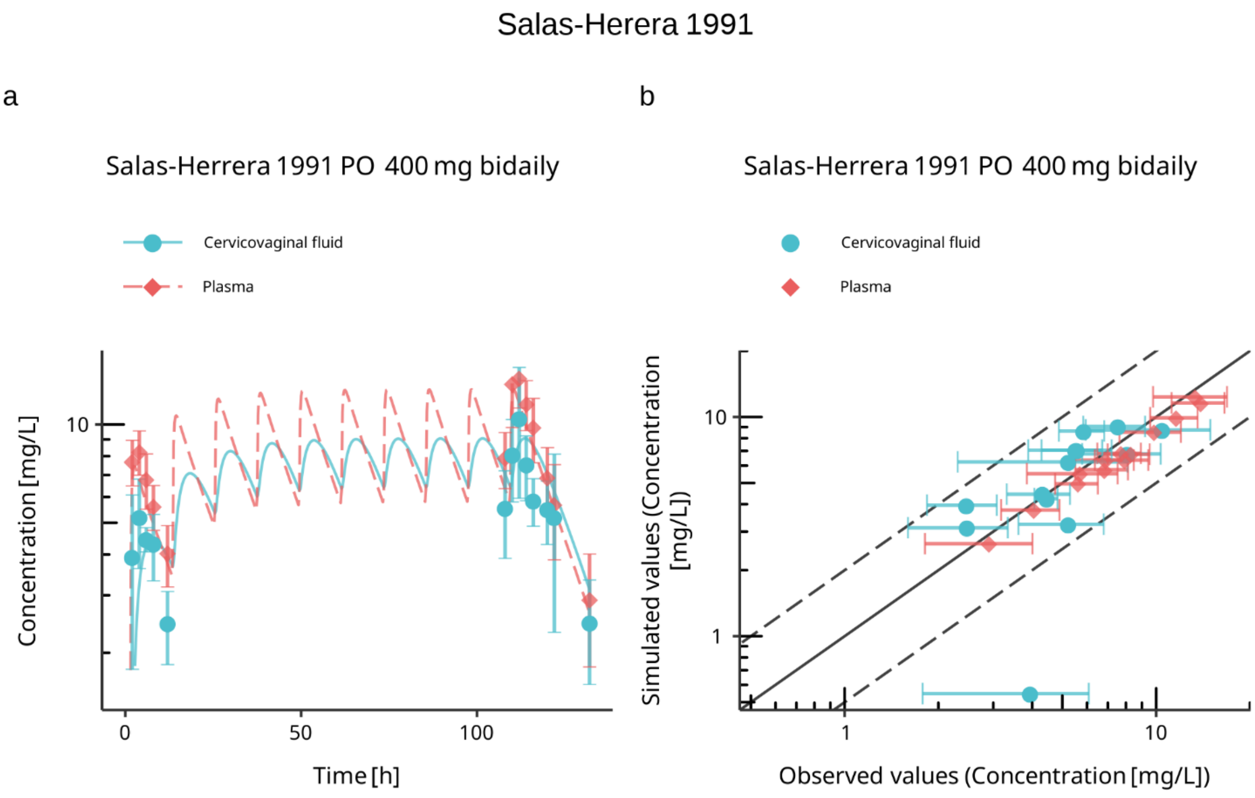

Figure 5: Model prediction for Salas-Herera et al. 1991 study.

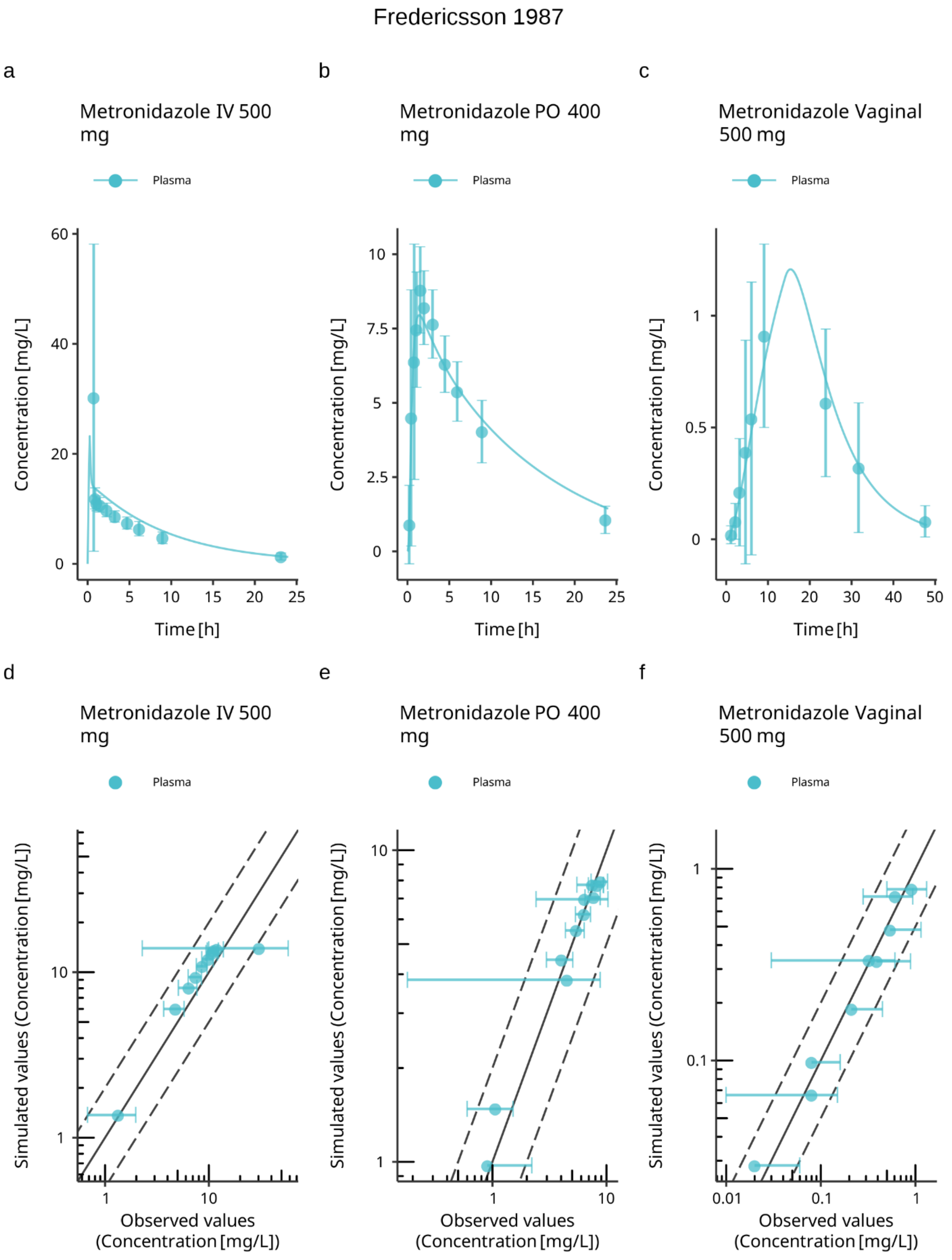

Figure 6: Model prediction for Fredericsson et al. 1987 study.

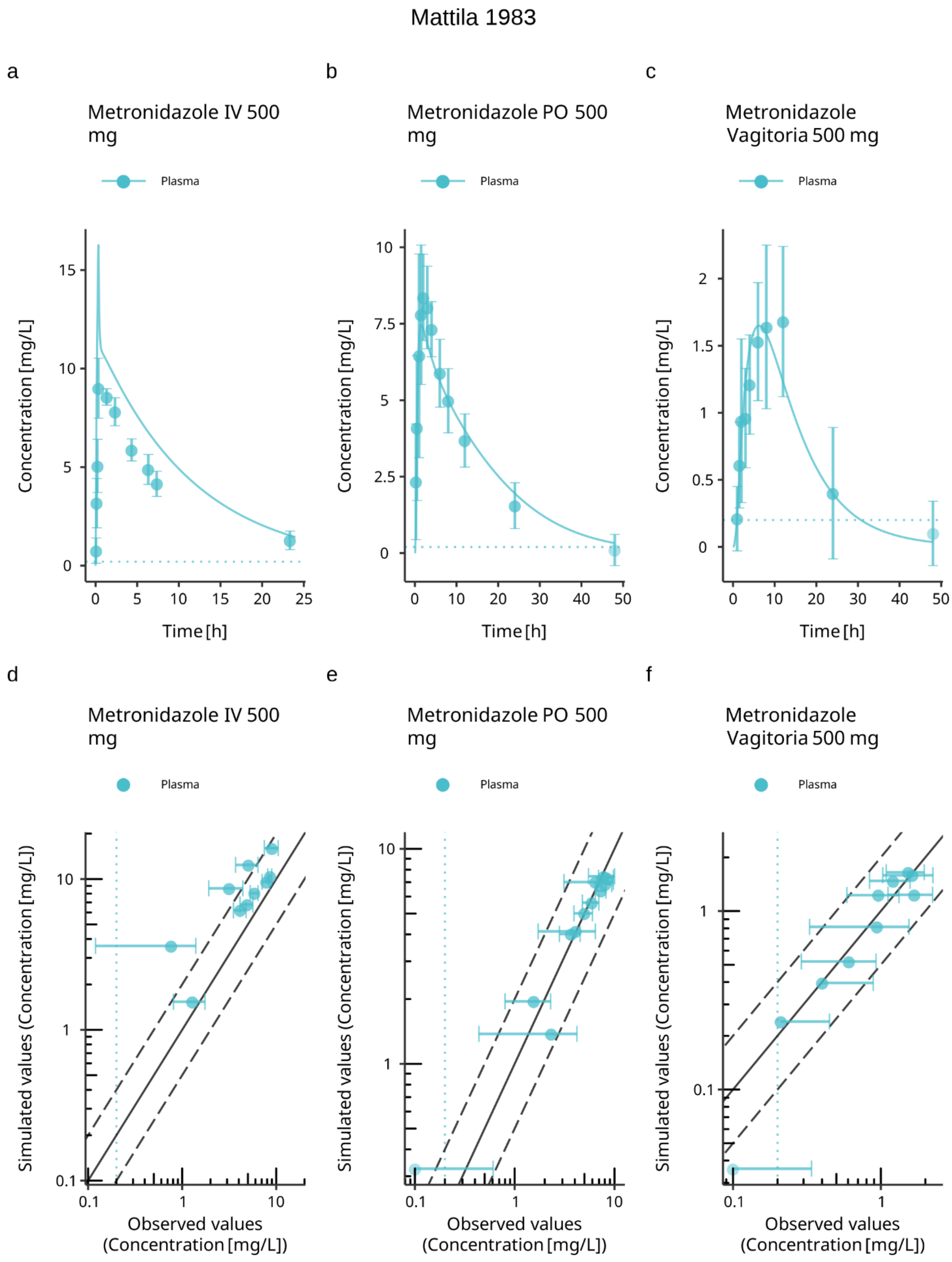

Figure 7: Model prediction for Mattila et al. 1983 study.

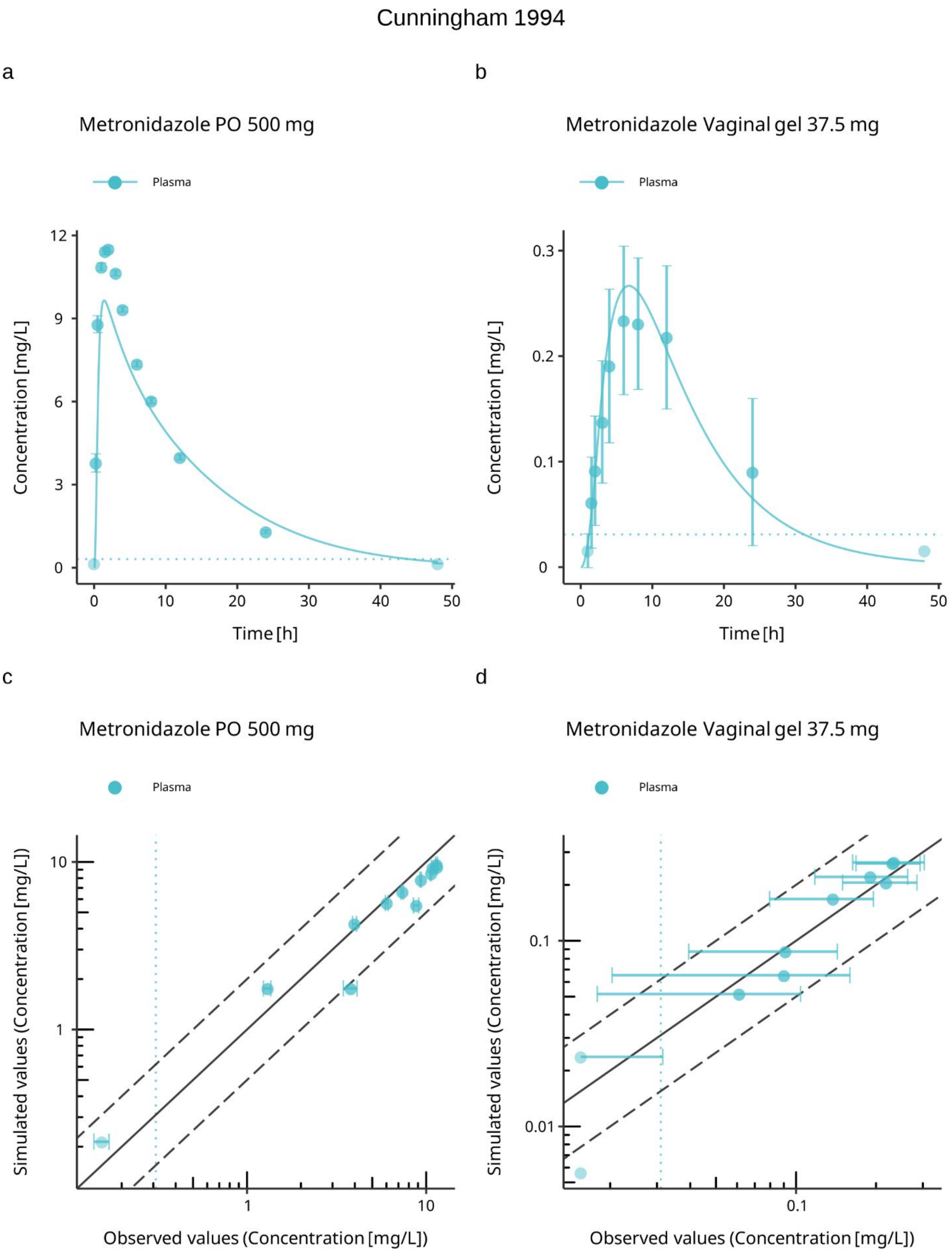

Figure 8: Model prediction for Cunningham et al. 1994 study.

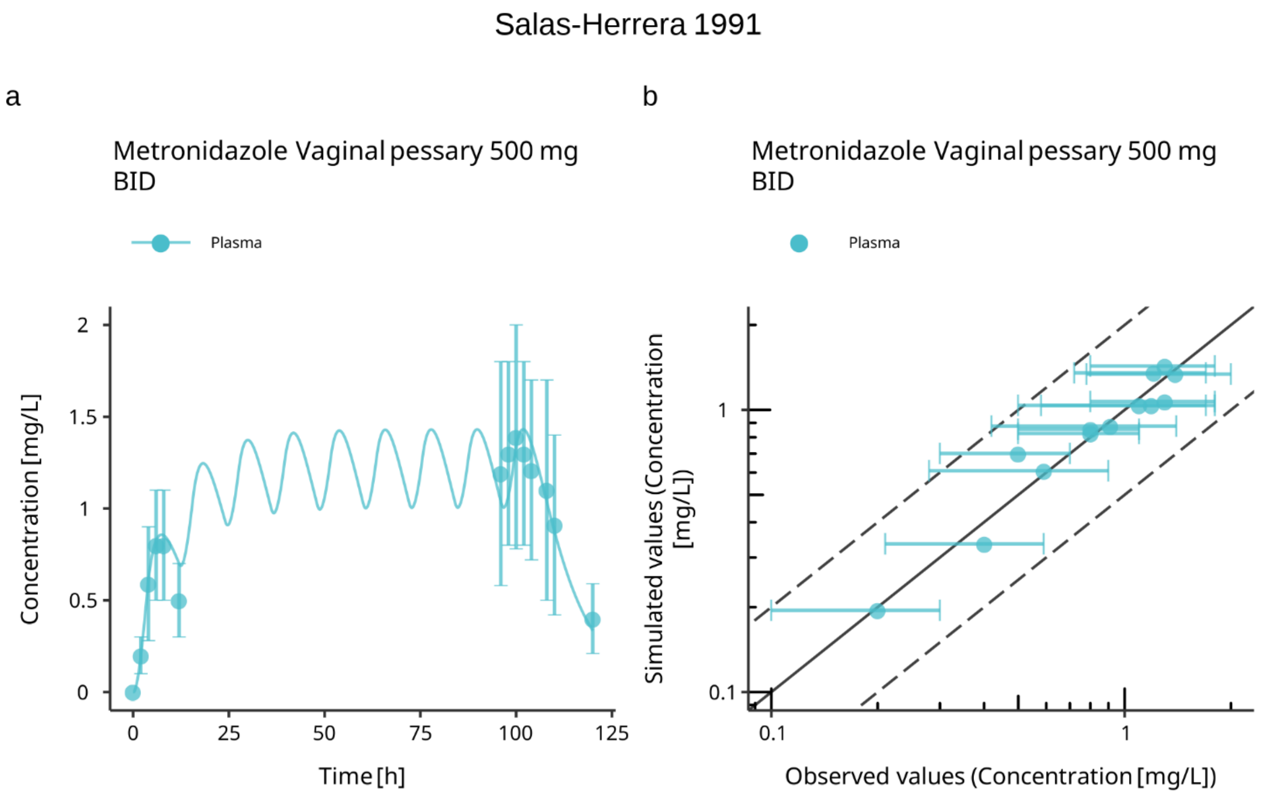

Figure 9: Model prediction for Salas-Herrera et al. 1991 study.

Table 6: Results of multiple start optimization -LNG.

run	Total error	scaling_endo start	scaling _endo	scaling_myo start	scaling_myo	D start [cm2/s]	D [cm2/s]	Note
1	0.84	0.11	0.11	1.81	0.23	1.03E-07	7.13E-06	converged
2	0.85	0.46	0.1	1.64	0.22	3.02E-08	1.90E-06	converged
3	0.85	0.29	0.1	0.95	0.22	3.20E-11	2.48E-06	converged
4	1.62	1.23	0.98	1.75	0.24	2.02E-10	1.55E-06	converged
5	0.84	4.47	0.1	3.97	0.23	8.58E-08	2.32E-06	converged
6	0.84	1.68	0.1	2.78	0.23	9.17E-11	1.32E-04	D at upper bound
7	0.84	0.46	0.11	0.81	0.23	1.06E-06	2.46E-06	converged
8	0.84	3.63	0.1	4.41	0.23	1.26E-07	3.21E-05	converged
9	0.85	0.27	0.1	0.14	0.2	4.48E-06	3.27E-06	converged
10	0.85	6.13	0.11	5.17	0.24	7.57E-08	2.30E-06	converged

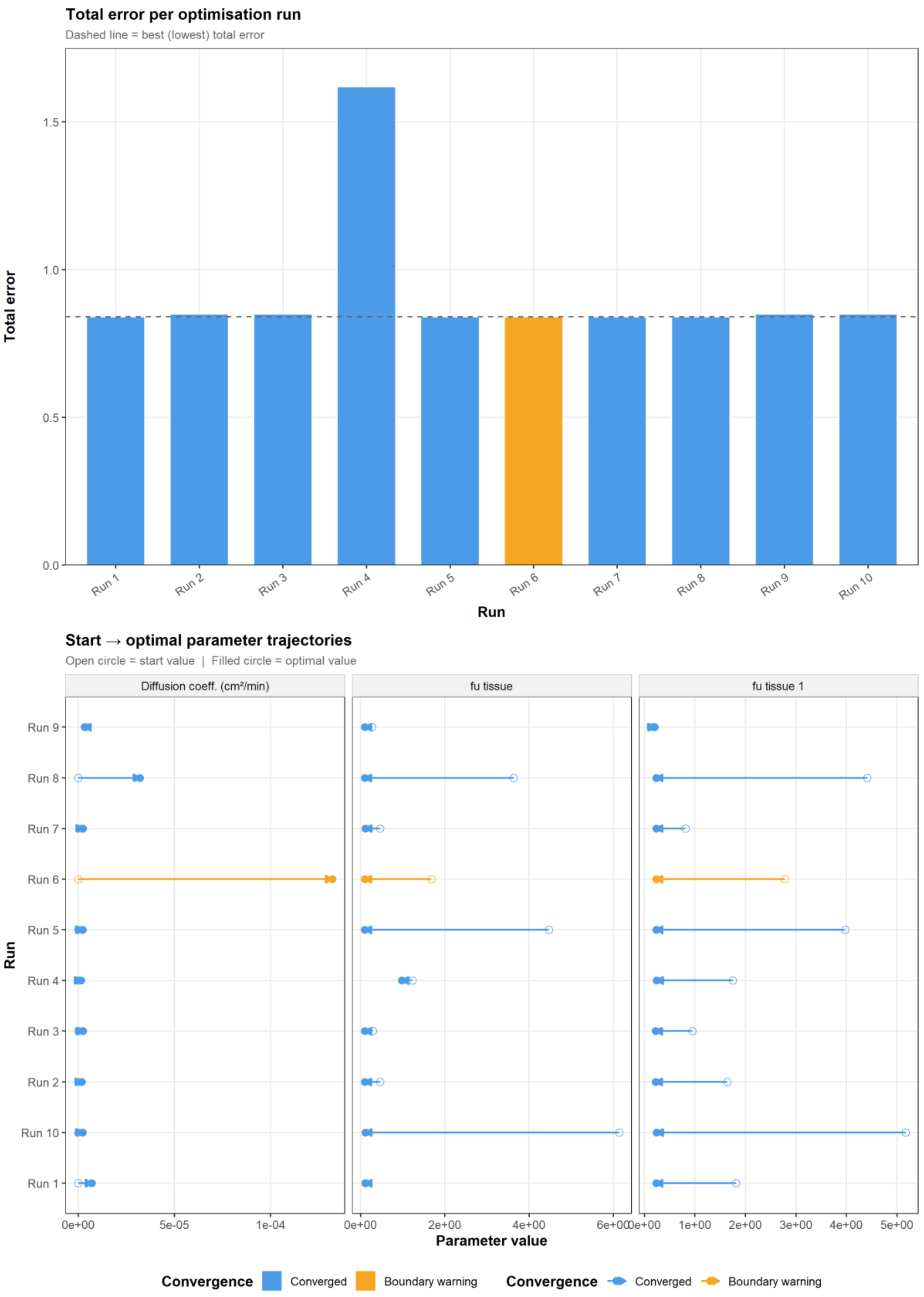

Figure 3: Visualization of multiple start optimization of LNG model (results presented in Table 7).

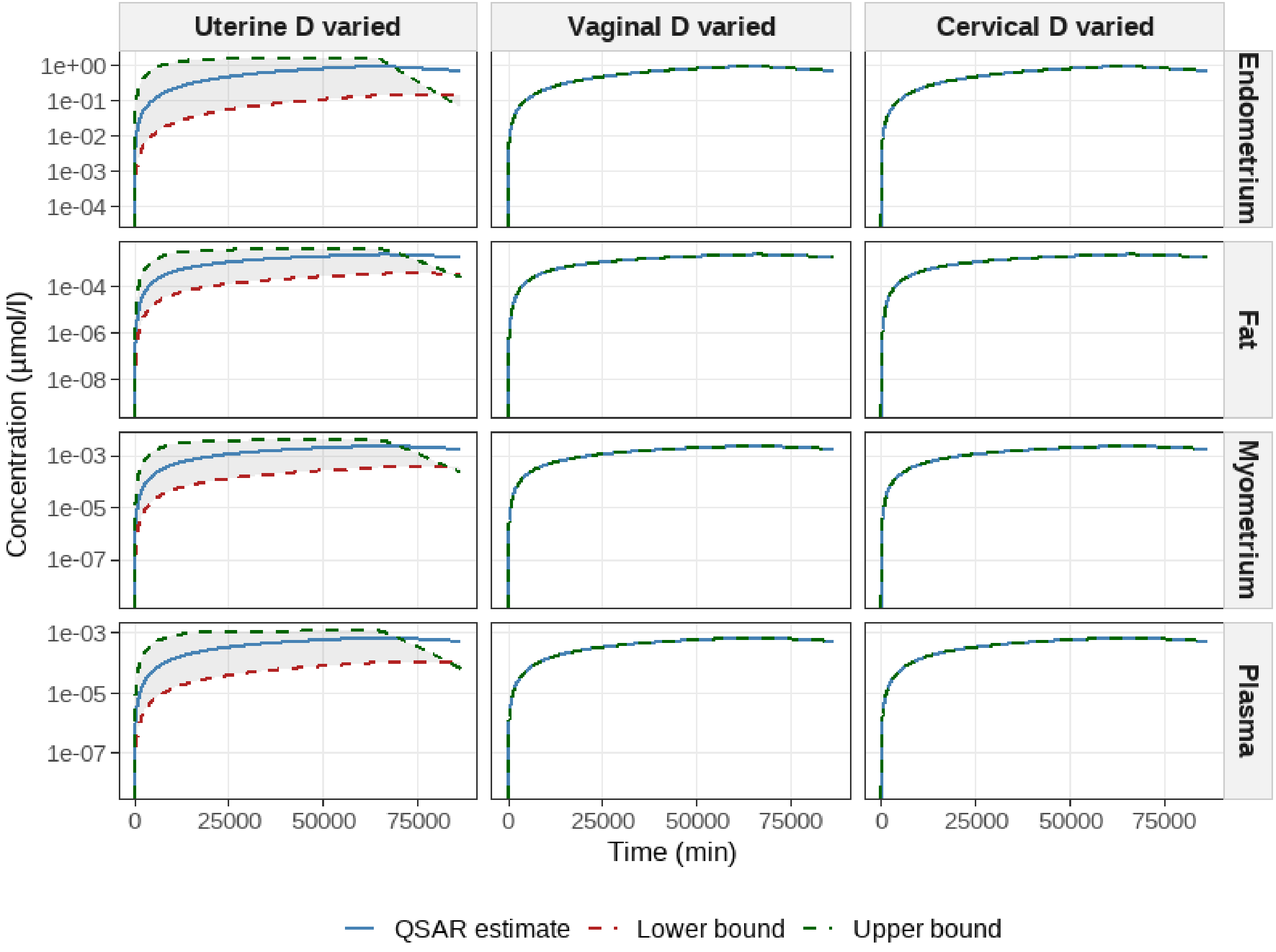

Uterine D varied
Vaginal D varied
Cervical D varied
Endometrium
Fat
Myometrium
Plasma
1e+00
1e-01
1e-02
1e-03
1e-04
1e-06
1e-08
1e-05
1e-07
Concentration (µmol/l)
0
25000
50000
75000
Time (min)
QSAR estimate
Lower bound
Upper bound

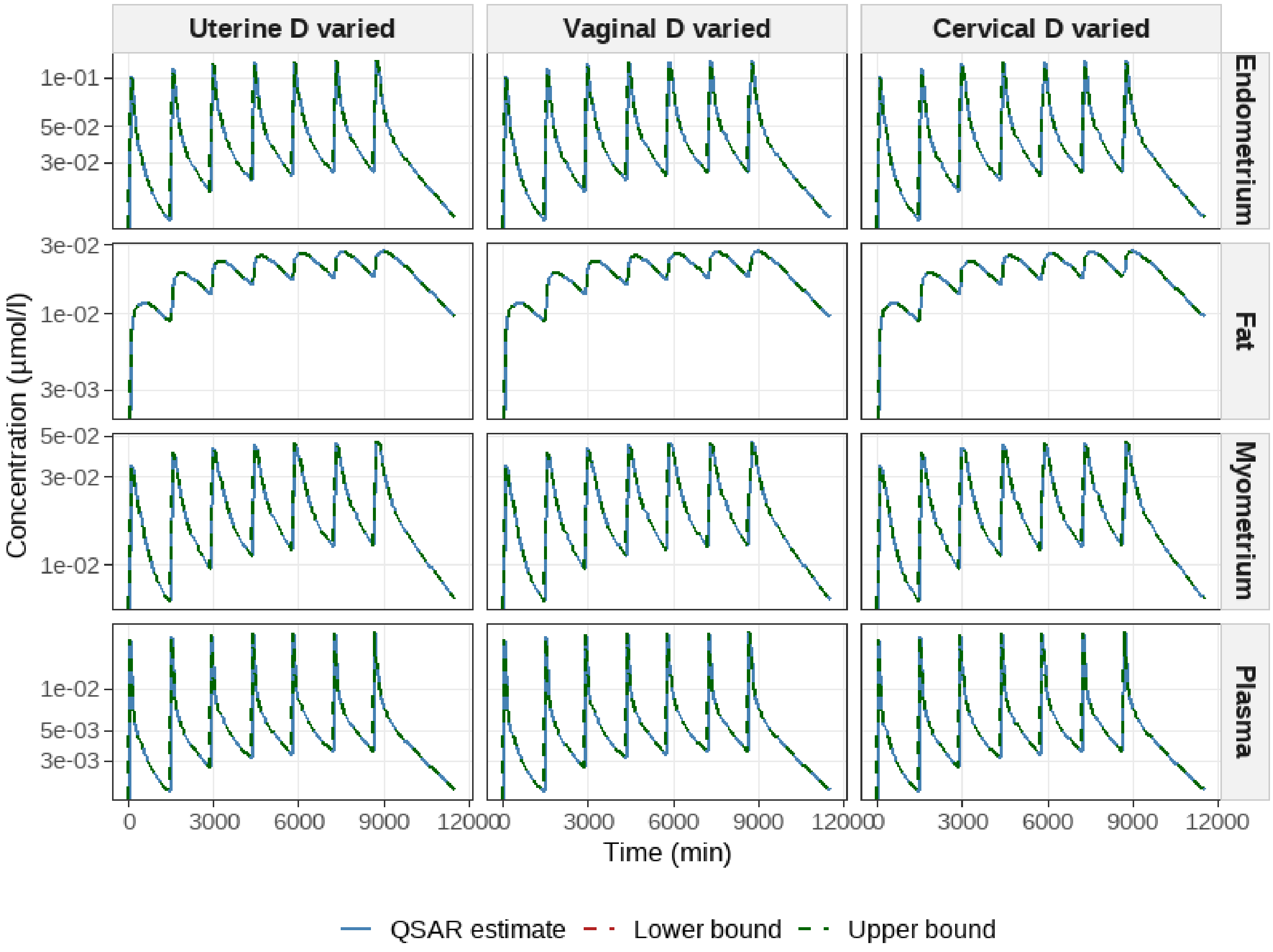

Figure 4: Bracketing uncertainty analysis for the uterine diffusion coefficient under oral administration. Simulated concentration-time profiles for the QSAR-derived diffusion coefficient estimate (solid blue) and ±1 $\log_{10}$ uncertainty bounds (dashed red: lower bound; dashed green: upper bound).

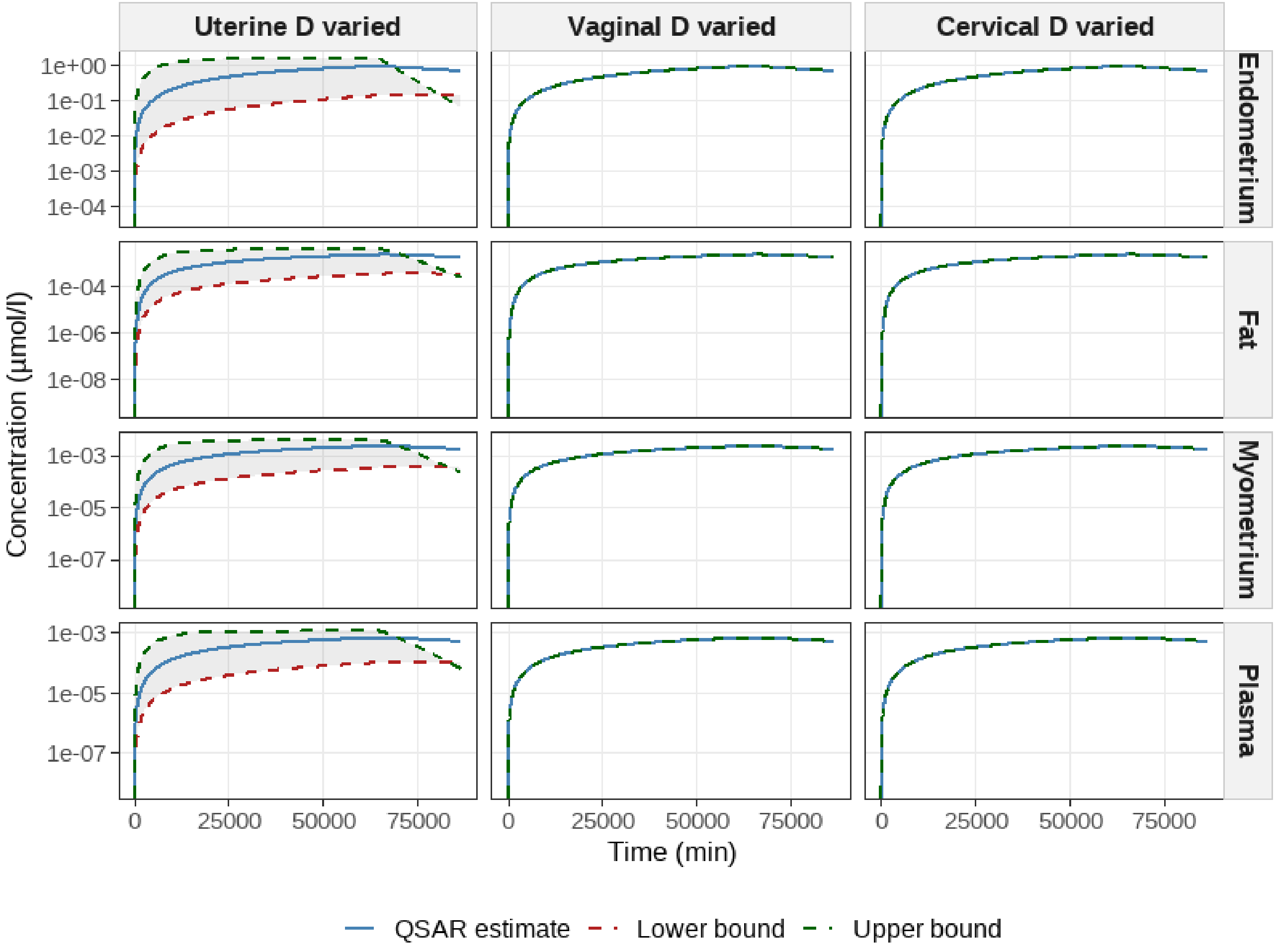

Figure 5: Bracketing uncertainty analysis for the uterine diffusion coefficient under intrauterine administration. Simulated concentration-time profiles for the QSAR-derived diffusion coefficient estimate (solid blue) and ±1 $\log_{10}$ uncertainty bounds (dashed red: lower bound; dashed green: upper bound).

Table 7: Tissue-to-plasma partition coefficients (Kp) for levonorgestrel (LNG) and metronidazole (MET) by FRT tissue compartment. Kp values are calculated using the Rodgers & Rowland method in PK-Sim/MoBi.

Compound	Tissue	Partition coefficient (intracellular/plasma)	Template tissue	Fitted/Fixed
LNG	Cervix	244.15	Muscle	Fixed
LNG	Endometrium	244.15	Muscle	Fixed
LNG	Myometrium	244.15	Muscle	Fixed
LNG	Vagina	244.15	Muscle	Fixed
MET	Cervix	0.7	Muscle	Fixed
MET	Endometrium	0.7	Muscle	Fixed
MET	Myometrium	0.7	Muscle	Fixed
MET	Vagina	0.7	Muscle	Fixed

Cicali, Brian, Karthik Lingineni, Rodrigo Cristofoletti, et al. 2021. "Quantitative Assessment of Levonorgestrel Binding Partner Interplay and Drug-Drug Interactions Using Physiologically Based Pharmacokinetic Modeling." *CPT: Pharmacometrics & Systems Pharmacology* 10 (1): 48–58. https://doi.org/10.1002/psp4.12572.

Cunningham, Francesca E., Donna M. Kraus, Linda Brubaker, and James H. Fischer. 1994. "Pharmacokinetics of Intravaginal Metronidazole Gel." *The Journal of Clinical Pharmacology* 34 (11): 1060–65. https://doi.org/10.1002/j.1552-4604.1994.tb01981.x.

Dallmann, André, Ibrahim Ince, Katrin Coboeken, Thomas Eissing, and Georg Hempel. 2018. "A Physiologically Based Pharmacokinetic Model for Pregnant Women to Predict the Pharmacokinetics of Drugs Metabolized Via Several Enzymatic Pathways." *Clinical Pharmacokinetics* 57 (6): 749–68. https://doi.org/10.1007/s40262-017-0594-5.

Fredricsson, Bengt, Bertil Hagström, Carl-Erik Nord, and Anders Rane. 1987. "Systemic Concentrations of Metronidazole and Its Main Metabolites after Intravenous Oral and Vaginal Administration." *Gynecologic and Obstetric Investigation* 24 (3): 200–207. https://doi.org/10.1159/000298803.

Männistö, P., H. Haataja, M. Karhunen, et al. 1984. "Concentrations of Metronidazole and Tinidazole in Female Reproductive Organs after a Single Intravenous Infusion and after Repeated Oral Administration." *Infection* 12 (3): 197–201. https://doi.org/10.1007/BF01640899.

Mattila, J, P T Männistö, R Mäntylä, S Nykänen, and U Lamminsivu. 1983. "Comparative Pharmacokinetics of Metronidazole and Tinidazole as Influenced by Administration Route." *Antimicrobial Agents and Chemotherapy* 23 (5): 721–25. https://doi.org/10.1128/AAC.23.5.721.

Pearce, Robin E., Michael Cohen-Wolkowiez, Mario R. Sampson, and Gregory L. Kearns. 2013. "The Role of Human Cytochrome P450 Enzymes in the Formation of 2-Hydroxymetronidazole: CYP2A6 Is the High Affinity (Low *K*m) Catalyst." *Drug Metabolism and Disposition* 41 (9): 1686–94. https://doi.org/10.1124/dmd.113.052548.

Salas-Herrera, Ig, M Lawson, A Johnston, P Turner, Dm Gott, and Mj Dennis. 1991. "Plasma Metronidazole Concentrations after Single and Repeated Vaginal Pessary Administration." *British Journal of Clinical Pharmacology* 32 (5): 621–23. https://doi.org/10.1111/j.1365-2125.1991.tb03962.x.

Salas-Herrera, L G, R M Pearson, A Johnston, and P Turner. 1991. *Concentration of Metronidazole in Cervical Mucus and Serum after Single and Repeated Oral Doses*.
